# Visual Agnosia and Posterior Cerebral Artery Infarcts: An Anatomical-Clinical Study

**DOI:** 10.1371/journal.pone.0030433

**Published:** 2012-01-20

**Authors:** Olivier Martinaud, Dorothée Pouliquen, Emmanuel Gérardin, Maud Loubeyre, David Hirsbein, Didier Hannequin, Laurent Cohen

**Affiliations:** 1 Department of Neurology, Rouen University Hospital, Rouen, France; 2 Inserm UMR-S 975 – CRICM, GH Pitié-Salpêtrière, Paris, France; 3 Université Paris 6, Faculté de médecine Pitié-Salpêtrière, Paris, France; 4 Department of Neuroradiology, Rouen University Hospital, Rouen, France; 5 Department of Ophthalmology, Rouen University Hospital, Rouen, France; 6 Inserm U 614, IFRMP, Faculty of Medecine and Pharmacy, Rouen, France; 7 AP-HP, Hôpital de la Salpêtrière, Department of Neurology, Paris, France; University of British Columbia, Canada

## Abstract

**Background:**

To evaluate systematically the cognitive deficits following posterior cerebral artery (PCA) strokes, especially agnosic visual disorders, and to study anatomical-clinical correlations.

**Methods and Findings:**

We investigated 31 patients at the chronic stage (mean duration of 29.1 months post infarct) with standardized cognitive tests. New experimental tests were used to assess visual impairments for words, faces, houses, and objects. Forty-one healthy subjects participated as controls. Brain lesions were normalized, combined, and related to occipitotemporal areas responsive to specific visual categories, including words (VWFA), faces (FFA and OFA), houses (PPA) and common objects (LOC). Lesions were located in the left hemisphere in 15 patients, in the right in 13, and bilaterally in 3. Visual field defects were found in 23 patients. Twenty patients had a visual disorder in at least one of the experimental tests (9 with faces, 10 with houses, 7 with phones, 3 with words). Six patients had a deficit just for a single category of stimulus. The regions of maximum overlap of brain lesions associated with a deficit for a given category of stimuli were contiguous to the peaks of the corresponding functional areas as identified in normal subjects. However, the strength of anatomical-clinical correlations was greater for words than for faces or houses, probably due to the stronger lateralization of the VWFA, as compared to the FFA or the PPA.

**Conclusions:**

Agnosic visual disorders following PCA infarcts are more frequent than previously reported. Dedicated batteries of tests, such as those developed here, are required to identify such deficits, which may escape clinical notice. The spatial relationships of lesions and of regions activated in normal subjects predict the nature of the deficits, although individual variability and bilaterally represented systems may blur those correlations.

## Introduction

Strokes in the territory of the posterior cerebral artery (PCA) constitute about ¼ of brain infarcts [1]. The range of clinical symptoms has been reported in several large series of patients but deficits were generally not assessed using systematic neuropsychological batteries [1], [2], [3], [4], [5], [6], [7]. Visual field loss is always the most commonly reported sign, with a frequency ranging from 84% [1], [3] to 100% [2]. Visual agnosia, often mentioned without further details, is much less frequent, ranging from 0% [1] to 3% [5]. This rate may rise to 8.5% in isolated infarctions of the superficial territory of the PCA [6]. Category specific agnosias are also reported: prosopagnosia occurs in 5.5% of cases at most [6] and pure alexia in 16% of cases [3].

Such low frequencies for visual agnosias may seem paradoxical, considering that functional imaging studies have evidenced an extensive and reproducible mosaic of occipitotemporal areas responsive to complex visual stimuli, with local preference for various categories of objects. Particularly, there are patches of cortex especially responsive to faces in the fusiform face area (FFA) [8], [9] and the occipital face area (OFA) [10], to words in the visual word form area (VWFA) [11], to body parts in the extrastriate body area (EBA) [12], to buildings and scenes in the parahippocampal place area (PPA) [13], and to common objects in the lateral occipital complex (LOC) [14]. So far, no distinct regions were identified for other classes of visual stimuli with the same degree of category specificity [15].

Moreover, the causal involvement of those regions in the identification of visual objects has been demonstrated by detailed case reports of brain-damaged patients. Thus prosopagnosia [16], [17], [18], pure alexia [19], [20], topographic agnosia [21] and deficit in various body-related tasks [22] have been attributed to lesions involving the FFA, OFA, VWFA, PPA, and EBA, respectively.

Considering the high overall frequency of PCA infarcts, such anatomical-clinical correlations would lead one to expect a relatively high frequency of agnosic deficits, a prediction which does not fit with the low rates reported in series of patients. Multiple reasons could contribute to such a discrepancy. *First*, some cerebral lesions affect only the earliest stages of visual processing (roughly up to V1/V2), inducing hemianopic scotomas, while sparing more anterior territories involved in high-level object recognition. Indeed, stroke registries report isolated occipital lobe involvement in as many as 60% of cases [6], and temporal artery involvement in only 39% of cases, most often in association with the calcarine artery [1]. *Second*, some actual but moderate agnosic deficits may be erroneously attributed to hemianopia. For instance, a careful study of reading latencies, eye movements, and lesion topography may be required to distinguish agnosic pure alexia from hemianopic alexia [19], [23], [24], [25]. Similarly, severe visual field defects may prevent agnosic patients from being adequately assessed and picked out in cohort studies of PCA lesions. This may be particularly true for patients with bilateral lesions, maybe a necessary condition for the occurrence of some types of agnosia [16]. *Third*, the circuits subtending visual analysis may be sufficiently redundant to prevent one single lesion to yield detectable deficits. For instance the PPA, as identified with functional imaging, is a roughly symmetrical structure. This may allow for effective functional compensation in case of unilateral lesions, which could in turn explain the infrequency of place recognition deficits. *Fourth*, patients may circumvent their deficit by resorting to alternative strategies developed, either spontaneously or through rehabilitation. For instance, in pure alexia, some patients develop effective letter-by-letter reading based on the operation of a network of intact areas [20], [26]. Similarly, prosopagnosic patients can identify people on the basis of indices other than facial configuration, and successfully engage in normal social interactions (for instance in developmental prosopagnosia [27], [28]).

As a consequence of redundant functional structures and of compensation strategies, a number of cases of mild agnosia may escape diagnosis, and require highly sensitive diagnostic tests. For example, normal scores in two commonly used face recognition tests, i.e. the Warrington Recognition Memory for Faces [29] and the Benton Facial Recognition test [30], do not necessarily demonstrate normal face recognition abilities [31]. Moreover, no standardized tests exist for other categories of visual stimuli, such as houses or body parts. Reading is not always systematically evaluated after a stroke and reading latencies, which may be necessary for the diagnosis of letter-by-letter reading, are often not recorded. Furthermore, most studies concerns fail to identify dissociations between categories, i.e. to assess the category specificity of agnosic deficits. Except in individual case-reports using multiple experimental tests, standardized visual neuropsychological tests generally do not ensure the comparability of the different visual stimuli, and resort to different tasks for different categories of stimuli.

The main aim of the present study was to evaluate systematically the cognitive and visual disorders following PCA infarctions, using a standardized battery including novel tests of visual recognition. These new tests should permit to detect slight visual deficits, and to determine whether those deficits are category specific. Moreover, lesion topography was correlated with cognitive performance using voxel-based lesion-symptom mapping (VLSM). The location of lesions was also compared to the average normal location of the VWFA, the FFA, the PPA and the LOC, as identified using functional MRI.

## Methods

### Ethics Statement

All patients gave their written consent after detailed information had been provided to them. The study was conducted in accordance with the Declaration of Helsinki, following approval by the ethics committee of the Hôpital de Bicêtre.

### Patients

From October 2005 to March 2009, a consecutive series of 31 patients with PCA strokes (ischemic or haemorrhagic) were recruited in the neurology department of the university hospital of Rouen, using the following criteria: age≥18 years, native speakers of French, normal mastery of the written language, no history of neurological or psychiatric disorder previous to the stroke, no drug abuse and no alcohol dependence, single lesion on CT or MRI scan. Only lesions involving the cortical territory of the PCA were selected while isolated thalamic and mesencephalic infarcts were excluded [32].

Among the 31 patients studied, 20 were men. All of them except one were right-handed according to the Edinburgh inventory [33]. The mean age was 57.7 years (range 20–80 years), and did not differ between left, right and bilateral strokes (p = .26; left: mean 52.3 years, range 20–75; right: mean 62.8, range 28–80; bilateral: mean 57.7, range 50–76). The mean educational level was 11.5 years (range 7–18 years), and did not differ across groups (p = .67; left: mean 11.3 years, range 7–17; right: mean 12.1, range 8–18; bilateral: mean 10.7, range 8–12). The neuropsychological study was conducted between 3.1 and 116.9 months after stroke with a mean delay of 29.1 months. The delay did not differ across groups (p = .78; left: mean duration 33.5 months, range 3.1–116.9; right: mean 26, range 3.2–93.4; bilateral: mean 20.5, range 6.8–43.7).

All patients had a detailed ophthalmic exam including visual acuity and visual field (Goldmann kinetic perimetry or automated Humphrey field analyser). Visual field defects were found in 23 patients (74%). Eight patients (26%) had no visual field deficit, nine (29%) had homonymous hemianopia and fourteen (45%) had quadrantanopia (lower 3 and upper 11).

### Cognitive assessment

Cognitive evaluation was conducted at least three months after the stroke. Patients were evaluated over two sessions, one for the standardized tests, and the other for all experimental tests.

#### Standardized tests

First, they underwent a series of background standardized tests evaluating non-visual functions, particularly memory and language: (a) the Mini Mental State Examination (MMSE) as a measure of global intellectual efficiency [34]; (b) an oral picture naming test (DO80) [35] and a shortened version of the Token test [36] to assess language, (c) forward and backward digit and spatial spans [37] as measures of working memory; (d) a French adaptation of the Grober and Buschke Verbal Learning test (GBVLT) [38] as a measure of verbal memory, the Doors test part A [39] and the recognition test from the Benton visual retention test [40] as measures of visual memory.

Second, they received standardized evaluations of visual function: overlapping figures of the ‘Protocole Montréal-Toulouse d'évaluation des gnosies visuelles’ (PEGV) [41], the Navon complex figures test [42] in which we presented 192 large letters made up of smaller letters, the test for matching objects different in viewpoint from the Birmingham Object Recognition Battery (BORB) [43], and the Benton judgment of line orientation test (JLOT) [44].

In addition to those standard tests, patients received 4 additional experimental tests designed to assess visual recognition: Picture detection in Array tests, Cambridge memory tests (CMT), Old/New discrimination tests and a timed word reading test. In the second session, the same order of assessment was used for all patients, taking a total of about 2 hours: Array test for houses, Old/New test for faces, Old/New sunglasses, CMT test with phones, Array words, Old/New flowers, Old/New scenes, Array faces, Old/New horses, CMT houses, Old/New cars, Array phones, Old/New tools, Old/New guns, CMT faces, Old/New houses, reading test. This order was chosen in order to avoid any repetition of the same visual category in successive tests.

#### Array test

**Figure 1:**
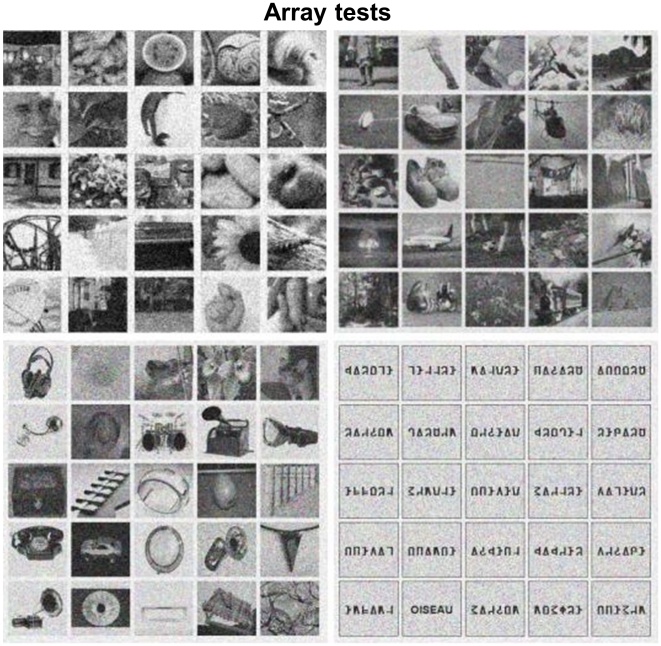
Examples of stimuli for the Array tests. For faces (upper left), houses (upper right), phones (bottom left) and words (bottom right). A target was present in the four presented examples.

Four versions of the Array test were used in order to evaluate the ability to detect an object from a given category in a crowded surrounding and under time pressure. This task might does not require a detailed item-specific representation, as categorization is sufficient to reach a decision in the detection task. We thus assume that the Array test reflects a rapid low-level visual processing. The reference version was developed by Brad Duchaine (University College London, Institute of Cognitive Neuroscience), using faces as the target category [45]. We created 3 parallel sets of arrays designed to test the perception of houses, phones and printed words. Each of the 4 tests included 50 different screens, each consisting of 25 images displayed as square arrays (Figure 1). One item of the target category (face, house, phone, printed word) was present in 50% of screens. For each version of the test, (a) the targets were 25 different exemplars from the target category; (b) the target appeared once in each of the 25 possible locations. The 4 versions of the test were run separately. The target category was first indicated to subjects. Then each screen was presented during 3 sec, and subjects were asked to decide as rapidly as possible whether it included a token from the target category, and to answer manually by pressing one of two keys. In all array tests, the target was hidden amongst distracter stimuli of comparable visual complexity. In the original array test for faces, which served as a “model”, distracters included a variety of items (objects, animals, fragments, etc). We followed the same principle for houses and phones, somewhat biasing the selection to ensure that spotting out targets would not be too easy. For instance, the distracters for phones included more graspable objects than the distracters for houses. For arrays in which the target was a printed word, ordinary pictures could not possibly be used as distracters. In order to achieve a comparable level of difficulty, we used strings of false-font letters. The Array Test for words used 25 upper case familiar nouns (mean log10 frequency per million between 2.01 and 2.79) and false fonts strings where each uppercase letter was replaced by an unfamiliar shape with an almost equal number of strokes and angles, and an similar overall visual appearance [46].

#### Cambridge memory test

**Figure 2:**
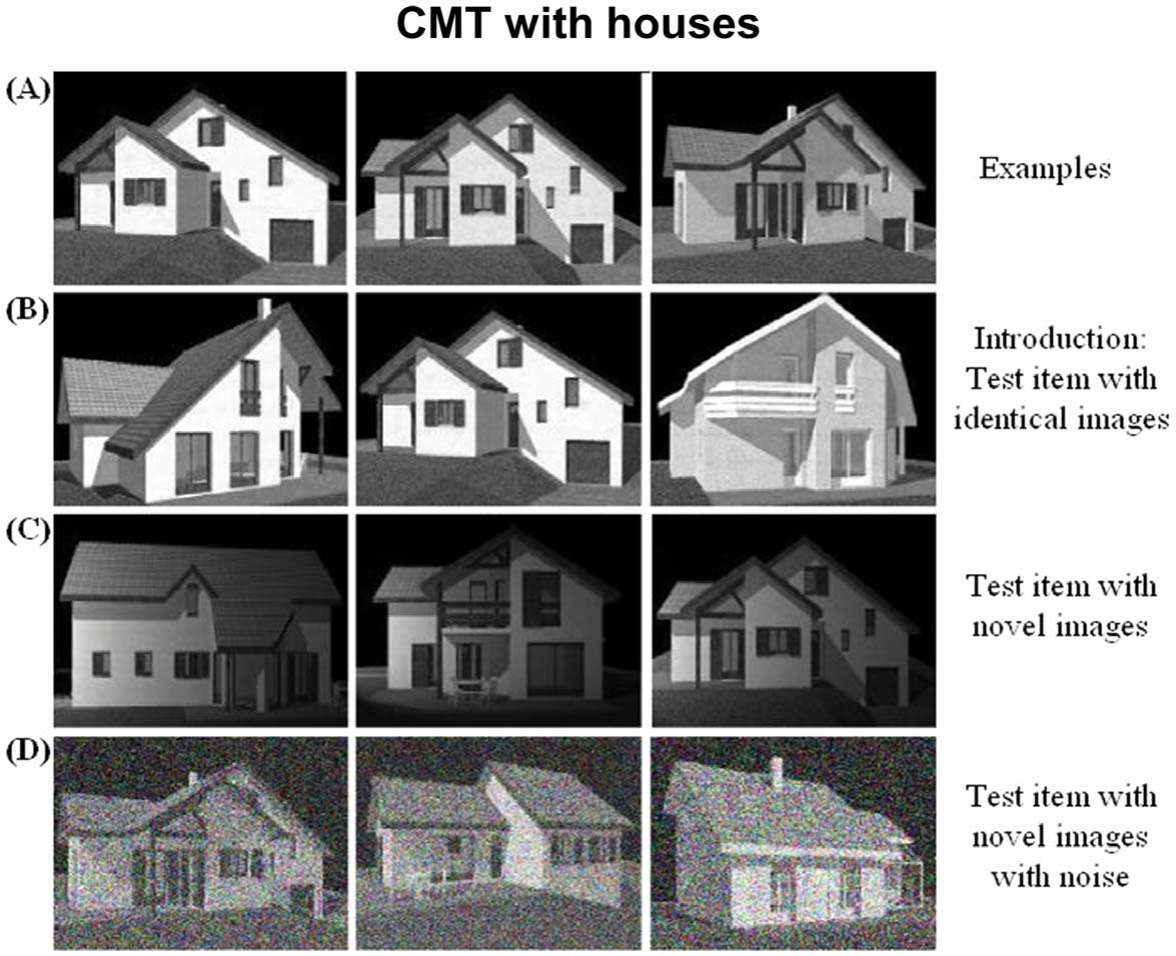
Examples of stimuli for the Cambridge Memory Test with houses. Panel A shows study views of a target house. Panel B displays a test item from the introduction. The central image is identical to the leftmost study view in Panel A. Panel C shows an item from the novel image section (the rightmost image is the target). Panel D displays a test item from the novel images with noise section (the leftmost image is the target).

**Figure 3:**
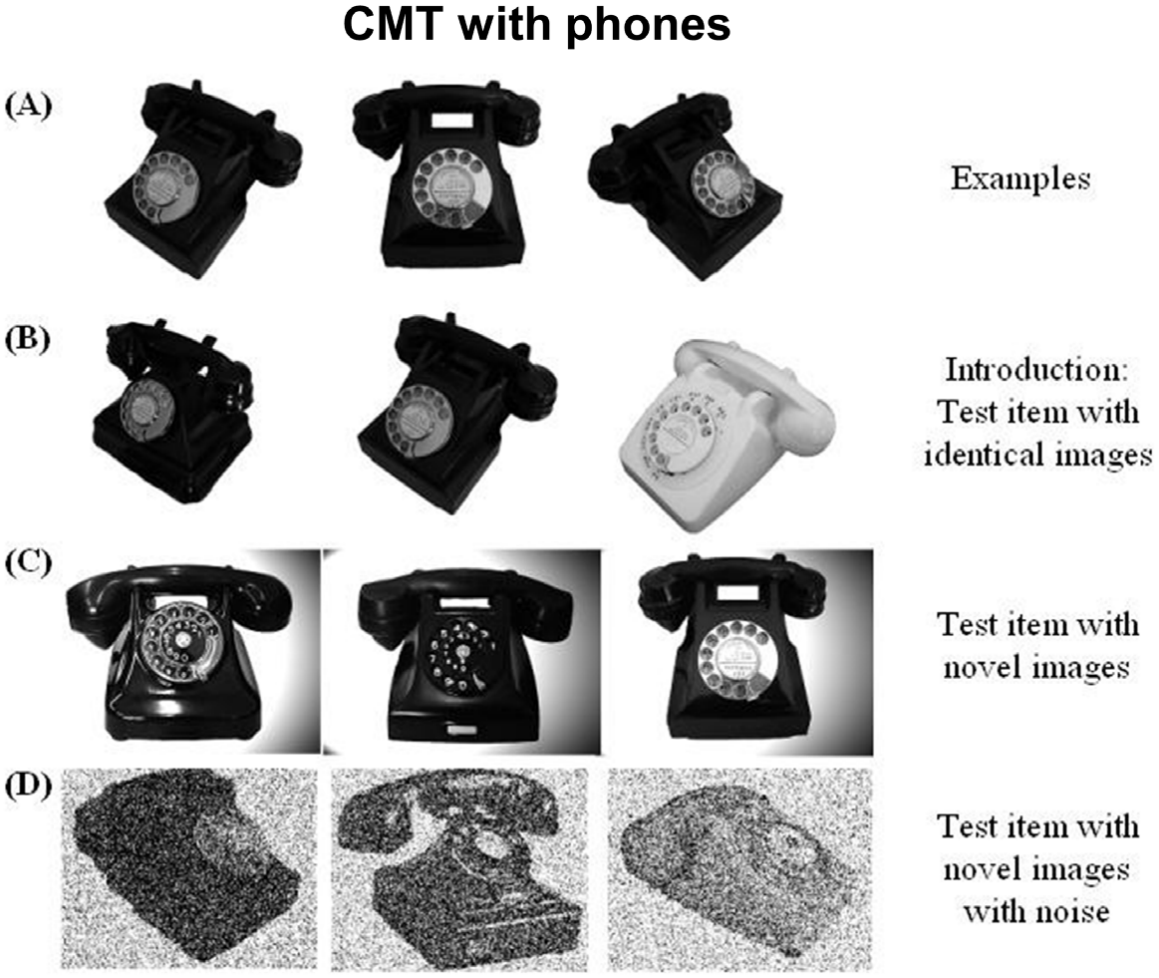
Examples of stimuli for the Cambridge Memory Test with phones. See legend of Figure 2.

The Cambridge Face Memory Test (CMT with faces) was originally designed to study the view-point processing of faces [47]. This test is highly sensitive to face processing deficits in prosopagnosic patients [47]. Most importantly, this test requires subjects to reach an invariant encoding of visual stimuli, in order to match pictures across changes in viewpoint or illumination. This property of invariance is thought to reflect the operation of occipitotemporal areas responsive to specific visual categories, such as faces (FFA) or houses (PPA) [48]. We derived parallel versions of this test, targeting houses and phones (Figure 2 and 3; Figure S1), in the goal to assess the same view-point specific properties of the PPA [49] and the LOC [50]. The houses were chosen on the website http://www.ideesmaisons.com/, which offers many different three-dimensional pictures in black and white. The phones were chosen from a variety of websites. For those two versions of the CMT, the six targets, as well as all the distractors, were selected with the same poses and lighting conditions. Distractors were used only once across the whole CMT procedure. Although they have not been formally published, all stimuli are available on request. The 3 versions of the test were run separately, in the same fixed order for all subjects (phones, houses, faces). The target category was first indicated to subjects. Each version of this test included four stages: practice, introduction/same images, novel images and novel images with noise. The practice stage was simply a familiarization to the procedure used in the introduction/same images stage. Three study images of one item of the target category were presented in succession, for 3 sec each. The study images included a left 1/3 profile, a frontal view, and a right 1/3 profile of the same item (see Figures 2 Panel A and 3 Panel A). Then, images of three different items from the target category were presented simultaneously, and participants were instructed to pick out the item corresponding to the study item, and to respond by pressing one of three keys (1, 2 or 3) (see Figures 2 Panel B and 3 Panel B). Importantly, at this stage, the target image to be selected was physically identical to one of the study images. This procedure was repeated for six target items. In the novel images stage, participants were first presented for 20 seconds with a review image combining frontal views of the 6 target items (Figure S1). They were then presented with 30 trials (6 target items×5 presentations) in a fixed, random order. Trials were analogous to those used at the previous stage: subjects had to choose among 3 images the one depicting any one among the 6 targets. However, contrary to the introduction/same images stage, target items were now presented with a novel lighting, pose, or both (see Figures 2 Panel C and 3 Panel C). This part of the CMT is therefore much more difficult than the previous one. Subjects are asked to recognize targets across changes in visual display, presumably resorting to more invariant representations; moreover, on each trial they may have to pick out any of the 6 target items. The last stage had exactly the same structure, except that it comprised only 24 test items (6 target items×4 presentations), and that fifty percent Gaussian noise was added to all the targets and distracters images (see Figures 2 Panel D and 3 Panel D).

#### Old/New test

Nine Old/New tests have been elaborated to evaluate the visual memory for 9 categories of objects: faces of women (with their hair masked), sunglasses, landscapes, flowers, horses, tools, cars, hand guns and houses [51]. On this test, prosopagnosic subjects show a disproportionate impairment with faces [28]. Moreover, using a similar task, Golarai et al showed that, across normal subjects, performance for faces and houses was positively correlated with the size of the right FFA and the left PPA, respectively, pointing to a sensitivity of this test to the function of specialized ventral visual cortex [52]. The 9 Old/New tests were run separately. First, participants were presented with ten target items of each category one at a time for 3 sec per item, and the ten targets were cycled through twice. During the test phase, participants were asked to respond whether an item was a target or a non-target as quickly as possible with a mouse click. A total of 50 test items of each category were presented consisting of 20 targets (10 targets×2 presentations) and 30 non-targets (30 non-targets×1 presentation).

#### Timed reading test

Finally, we used a timed word reading test. This test allows for the detection of reading errors, but also of the letter-by-letter reading strategy typically used by patients with pure alexia. We used 165 lower-case familiar nouns (mean log10 frequency per million = 2.4), 3–9 letters and 1–4 syllables in length [19]. They were presented centrally on a computer screen, subtending a maximum angle of 4° on each side of fixation. Subjects were asked to read each word aloud rapidly, while minimizing errors. Stimuli remained visible until a response was produced. Latencies were measured using a voice key, and sessions were recorded for subsequent scoring of errors.

### Control subjects

Forty-one healthy subjects (mean age 50.8 years [range 20–78 years]; mean educational level 13.9 years [range 7–28]; 16 men) participated in the study. They performed a MMSE (mean score 28.9 [range 26–30] and the experimental tests (Array, CMT, Old/New, and word reading). They all gave their written informed consent. Mean age (p = .12), mean educational level (p = .09) and sex (p = .11) did not differ between control subjects, left PCA and right PCA strokes. Mean score of the MMSE for control subjects was better than left PCA strokes (p = .022) but did not differ from right PCA strokes (p = .20).

### Statistical analysis

#### Standardized tests

Performance was considered abnormal below the 5^th^ percentile of normative data adjusted for age and level of education. Verbal memory was considered as impaired when at least one score of the Grober and Buschke Verbal learning test was abnormal [38]. Visual memory was considered as impaired when the score of the Doors test part A [39] or of the recognition score of the Benton visual retention test [40] was abnormal. Visual processing was considered as impaired when at least one score among the PEGV [41], Navon [42], BORB [43], and JLOT [44] was abnormal.

#### Experimental tests

For each experimental test we first performed group analyses on the raw data using ANOVAs and post-hoc test of Newman-Keuls (STATISTICA 7.1 software), with group (controls, left-hemisphere patients, right-hemisphere patient) as between-subjects factor, stimulus category as within-subjects factor, and subjects as random factor. The 3 subjects with bilateral lesions were excluded from this group analysis.

In a second stage, the individual patient's performance on each test was converted to a z-score based on the mean and the standard deviation (SD) of the control sample. Performance was considered abnormal when the z-score was ≤−2. To identify *selective* deficits we used the stringent Revised Standardized Difference Test (RSDT) criterion [53]. To be considered category-specific for faces, houses, etc, a deficit should fulfil a double requirement: (i) only one category should be affected across all tests; and (ii) the impairment should be significantly greater for this category than for all others categories, within each of the relevant tests (Arrays, CMT, Old/New). This second criterion is assessed using a modified t-test allowing for the comparison of one patient's score against a group of controls of moderate size.

### Analysis of brain imaging

Anatomical T1-weighted MRI images from 24 out of 31 patients were linearly transformed into MNI space (standard template of the Montreal Neurological Institute) using the SPM software. Lesions were drawn manually, slice by slice, on the native image, then smoothed (4 mm FWHM Gaussian filter), thresholded (>0.5), and normalized with the same matrix as anatomical images. In the remaining 7 patients, only CT-scan images were available, and lesions were drawn on a brain template in MNI space. Lesions were studied in three manners.

First, we studied their spatial relationship with the average peak location of specialized visual areas, as defined on the basis of published imaging studies: the FFA [40,−55,−10 and −35,−63,−10] [9], the OFA [37,−74,−17 and −36,−73−,17] [54], the VWFA [−44,−58,−15] [55], the PPA [30,−44,−14 and −27,−46,−15] [56] and the LOC [45,−76,0 and −39,−73,0] [57]. Spherical regions of interest (10 mm radius) were centered on each peak, and their overlap with lesions analysed.

For the two other methods of lesions analyses, which were applied separately for the 3 main categories of stimuli, the patient's performance was coded binarily. For each of the three main categories of stimuli (faces, houses and words), patients were categorized as impaired if they had a pathological performance on at least one experimental test (i.e. arrays, CMT, Old/New tests and reading task). In the second type of analysis, regions of maximum overlap were identified by adding lesions, with a positive weight for patients with a deficit and an equal negative weight for patients without a deficit [19]. The third type of analysis consisted in voxel-based lesion-symptom mapping (VLSM) [58], which allow to compare the performance of patients with and without a lesion of each voxel, with voxelwise statistics corrected for multiple comparisons over the whole brain (threshold: FDR p<0.05) (NPM package and MRIcron software, http://www.cabiatl.com/mricro/).

## Results

### Lesion topography

**Figure 4:**
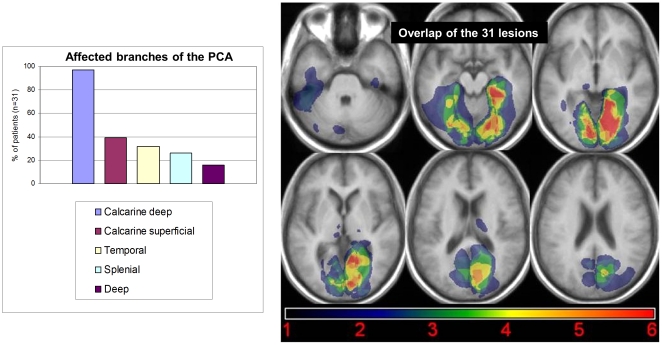
Vascular topography of strokes, and overlap of the 31 lesions. Vascular topography of strokes (left panel A), and overlap of the 31 lesions in MNI space (slices are TC z = −33, −15, −5, 4, 16, 24; right panel B).

Lesions were located in the left hemisphere in 15 patients (48%), in the right in 13 (42%), and bilaterally in 3 (10%). PCA territory is summarized in Figure 4. In 26 patients (84%), strokes were limited to the cortical territory while 5 patients (16%) had cortical and deep infarcts. The calcarine artery territory was always affected, including one case with an isolated infarct of the superficial cortical branches, and 12 cases with isolated deep cortical branches infarcts. The temporal artery territory was affected in 10 cases and the splenial artery territory in 8, always with calcarine artery involvement.

### Standardized cognitive assessment

Neuropsychological findings (global efficiency, language, memory, and visual abilities) in the 31 patients are summarized in Table S1. MMS was below the cut-off score in three patients suffering a left stroke. Two patients with a left infarct had a pathological naming score of the DO80, but no patients had a significant comprehension deficit on the Token test. Memory was impaired in 16 patients: 5 had verbal memory deficit, 5 visual memory deficit and 6 had both.

Fifteen (48%) patients had a visual disorder, 6 without any other cognitive deficit, 9 with an associated memory deficit (2 visual, 2 verbal, 5 both). The JLOT was by far the most frequently impaired test (13 patients out of 15).

### Group analysis of the experimental tests

The results of the experimental study in patients and controls are reported in Table S2 (Array tests, Cambridge Memory Tests and Old/New Tests), and Table S3 (Reading test). Twelve patients (39%) had a deficit on at least one Array test, 11 patients (35%) on at least one Cambridge test (Figures S3, S4, S5), and 11 patients (35%) on at least one Old/New test. Overall, 20 patients (65%) had a deficit on at least one of those tests.

#### Array tests

**Figure 5:**
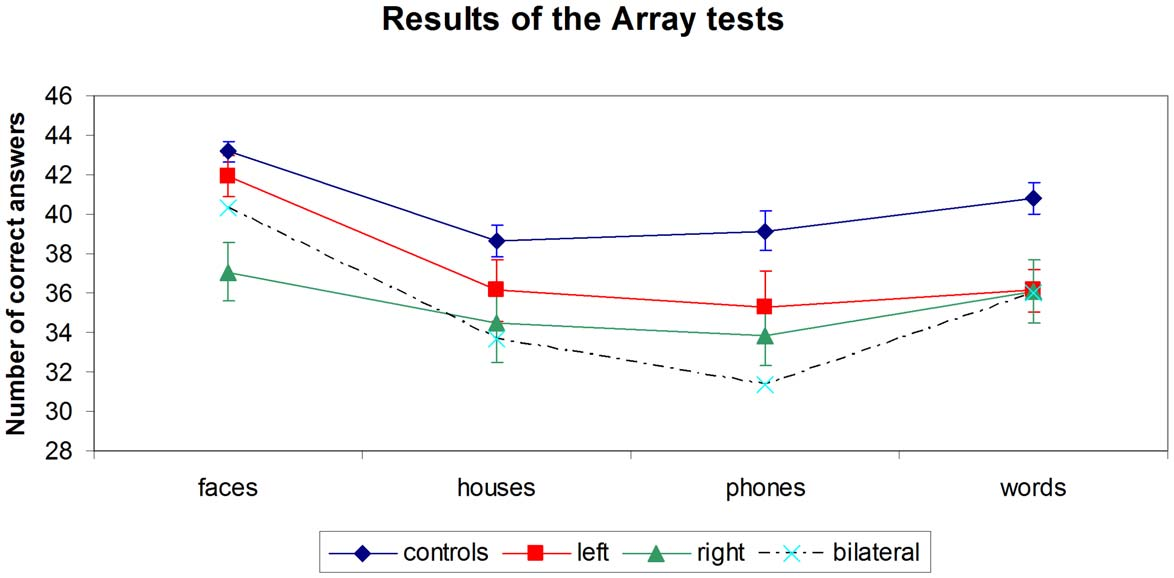
Results of the Array tests for faces, houses, phones and words. In controls (n = 41), left stroke patients (n = 15), right stroke patients (n = 13) and bilateral stroke patients (n = 3). Error bars represent +/−1 S.E.M.

In control subjects, the rate of correct responses differed across types of stimuli (F(3,120) = 20; p<.001; Figure 5). Performance was better for faces (86% correct) than for words (82% correct; p<.001), and better for words than for houses (77% correct; p<.002) and phones (78% correct; p<.017), which did not differ.

Patients showed the same overall profile (Figure 5). There was an effect of stimulus type (F(3,90) = 14.4; p<.001), and performance was again better for faces (79% correct) than for the three other types of stimuli (ps<.001), which did not differ (72%, 70%, and 69% correct for words, houses and phones, respectively).

We then compared controls versus left and right unilateral patients. Averaging across stimulus types, patients performed worse than controls (F(2,66) = 7; p<.002), with no overall difference between left and right patients (p = .28). There was a marginal interaction of lesion side x stimulus type (F(3,78) = 2.3; p = .08). As suggested by Figure 5, left and right patients did not differ for houses, phones or words (ps>.05). For faces, right patients performed worse than left patients (p = .011), who did not differ from controls (p = .25).

In summary, patients performed overall worse than controls, with a more salient impairment for faces in right-hemisphere patients.

#### CMT

For each type of stimuli, the test included 3 parts. The first part probed mainly immediate visual recognition, while parts 2 and 3 tested more abstract and more differed recognition. Indeed, with each of the three types of stimuli, performance in parts 2 and 3 were strongly correlated across subjects (r = .77, p<.001; r = .45, p<.01; r = .66; p<.001), while their correlation with part 1 was weaker. We therefore averaged the data of parts 2 and 3 (full data are provided in Table S4).

**Figure 6:**
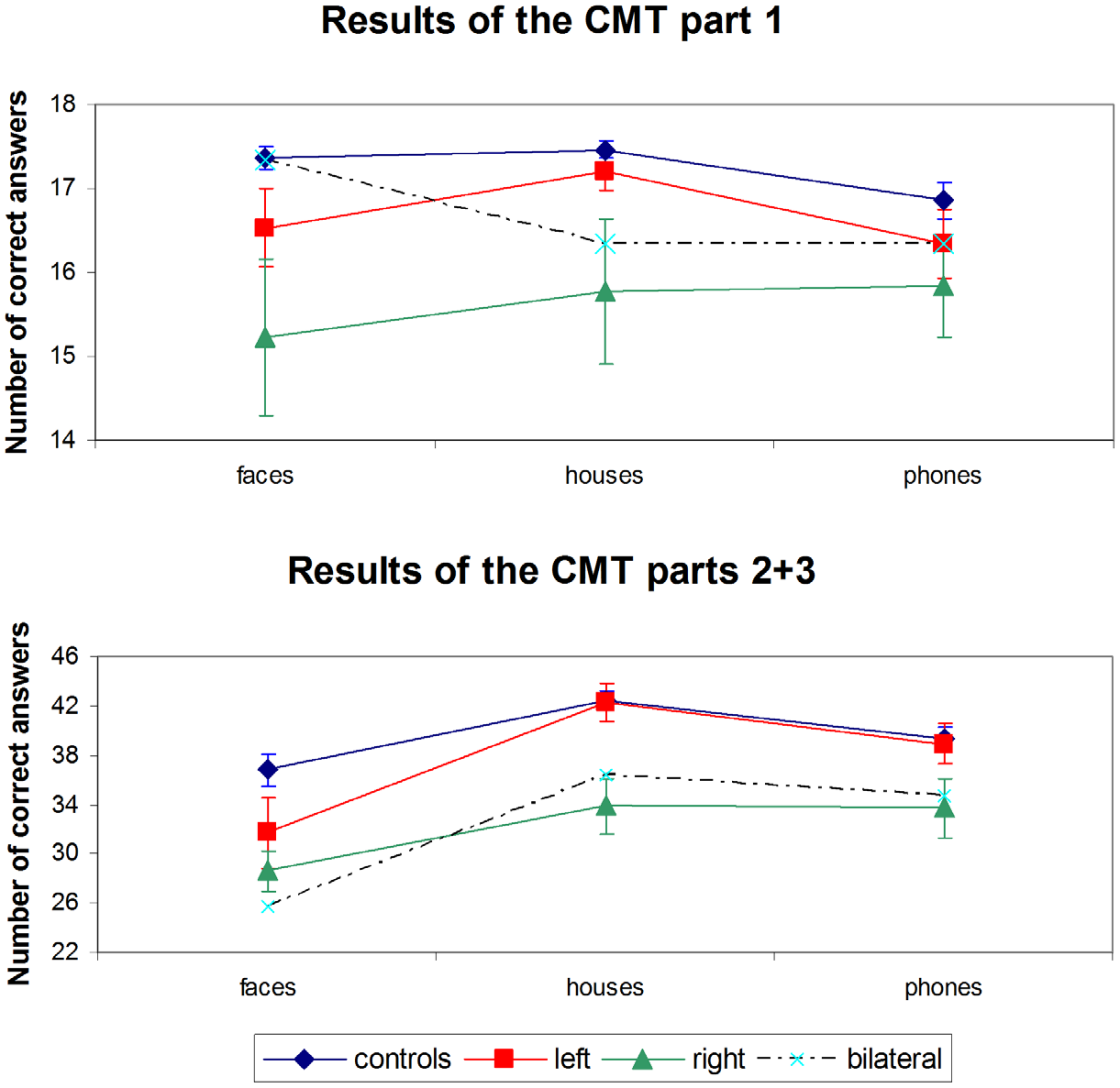
Results of Cambridge Memory tests with faces, houses and phones. In controls (n = 41), left stroke patients (n = 15), right stroke patients (n = 13) and bilateral stroke patients (n = 3). Error bars represent +/−1 S.E.M.

In control subjects, there was an interaction of stimulus type and test part (F(2,80) = 9.9; p<.001; Figure 6). In part 1, there was a moderate but significant effect of type (F(2,80) = 4.2; p = .018), with better performance for faces (96% correct) and houses (97% correct) than for phones (94%). In part 2/3, the differences across stimulus types was much bigger (F(2,80) = 11.5; p<.001), with better performance for houses (78% correct) than for phones (72% correct) than for faces (68% correct).

Patients showed the same interaction of stimulus type and test part (F(2,60) = 12.3; p<.001; Figure 6). In part 1, there was no effect of type (p = .5). In part 2/3, there was a strong effect of category (F(2,60) = 22.2; p<.001), with the same profile as in controls, i.e. better performance for houses (70% correct) than for phones (67% correct) than for faces (55% correct).

We then compared controls versus left and right unilateral patients. Averaging across stimulus types, patients performed worse than controls (F(1,67) = 12.9; p<.001, and F(1,67) = 9.4; p = .003, for parts 1 and 2/3, respectively), and left patients performed somewhat better than right patients (p = .04 and p = .08, for parts 1 and 2/3, respectively). There was no interaction of lesion side x stimulus type (p = .86 and p = .36, for parts 1 and 2/3, respectively).

In summary, right-hemisphere patients performed worse on the CMT than controls and left-hemisphere patients, across all categories of stimuli.

#### Old/New tests

**Figure 7:**
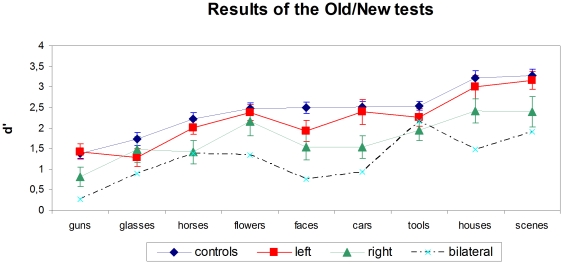
Results of Old/New tests. In controls (n = 41), left stroke patients (n = 15), right stroke patients (n = 13) and bilateral stroke patients (n = 3). Error bars represent +/−1 S.E.M.

For each subject and each category of stimuli, a d′ was computed as an unbiased measure of the ability to discriminate old versus new stimuli. d′ values were entered in ANOVAs with subjects as random factor, category of stimuli as within-subject factor, and group as between-subject factor. Subjects with bilateral lesions were excluded from this group analysis. As visible on Figure 7, performance varied across categories (F(8,520) = 47.9; p<10^−3^), scenes and houses being the easiest to discriminate, and guns and glasses the most difficult. There was also a main effect of group (F(2,65) = 5.26; p = .0076). Right-hemispheric patients performed worse than controls (F(1,51) = 9.9; p = .0028), marginally worse than left-hemispheric patients (F(1,25) = 2.78; p = .10), while the two latter groups did not differ (F(1,54) = 1.6; p = .22). There was no significant interaction between group and category.

Again, right-hemisphere patients preformed worse on the Old/New tests than controls and left-hemisphere patients, across all categories of stimuli.

### Individual results of the experimental study

As mentioned before, 20 patients (65%) were significantly impaired in at least one score. Across subjects, such abnormal scores concerned all types of tests, and all the main categories of stimuli (see Tables S2 and S3).

#### Selective deficits

Pooling all tests except the timed reading test, six patients (#1, 2, 6, 16, 20, 29 see Table S2) had a deficit only for one single category of stimuli in one type of test. Patient #1 (left lesion) was impaired only on the CMT with houses, while he performed normally on the CMT with faces and phones. Note that according to the stringent RSDT statistical criteria, this patient was the only one to suffer from a truly selective deficit (CMT houses versus faces and houses versus phones: t(40) = 3.062; p<.01, and t(40) = 3.473; p<.01, respectively). Among the other 5, two patients also had a deficit for houses (#16, right lesion; #29, bilateral lesion); one for faces (#20, right lesion), one for words (#6, left lesion), and one for phones (#2, left lesion).

We then considered each type of tests separately (Arrays, CMT, or Old/New tests). Nine further patients (#10, 11, 12, 19, 21, 22, 24, 26, 31 see Tables S2 and S3) had a deficit for a single category of stimuli within one type of test, with additional deficits in other tests. Among them, six patients were impaired on one Array test (3 with faces: #19, 26, 31; 2 with houses: #10, 22; 1 with phones: #11), four on one CMT (1 with faces: #12; 1 with houses: #19; 2 with phones: #24, 26), and five on one Old/New tests (2 with faces: #12, 21; 1 with tools: #11; 2 with horses: #19, 24). According to the RSDT statistical criteria, two of those patients had a truly selective deficit. Patient #11 (left lesion) had a selective deficit for phones on the Array test (phones versus faces, phones versus houses and phones versus words: t(40) = 2.908; p<.01, t(40) = 2.014; p = .05, and t(40) = 2.851; p<.01 respectively), but also a deficit on the Old/New test for tools (not significantly different from the other Old/New tests). Patient #26 (right lesion) had a selective deficit for faces on the Array test (faces versus houses, faces versus phones and faces versus words: t(40) = 3.375; p<.01, t(40) = 2.238; p = .03, and t(40) = 2.094; p = .04 respectively), but also a deficit on the CMT with phones (not significantly different from the CMT with faces and houses).

#### Word reading

Two patients (#8, 9, left lesions) were moderately but significantly impaired on the word reading test (155/165 and 157/165 correct responses, respectively; Table S3), with no word length effect. They performed normally on other experimental tests. A third patient (#12, left lesion) had a more severe reading impairment (142/165), with a word length effect (315 msec per letter). He showed associated deficits in the Array tests for faces and houses, the CMT with faces and the Old/New test with faces.

In summary, about one third of impaired patients had a deficit selective for one category of items. We now turn to an analysis of brain lesions, trying to determine, for each category of deficit, the region of maximum overlap of lesions and the correlations of lesions with the average location of specialized visual areas as identified with functional imaging.

### Anatomical-clinical correlations

**Figure 8:**
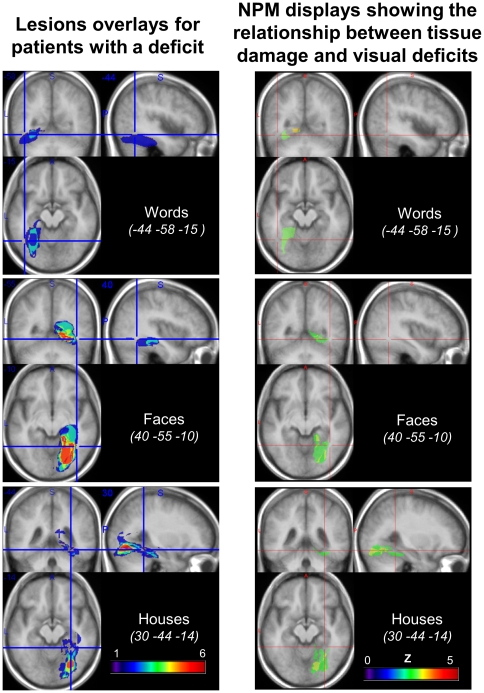
Lesion analysis. Voxel-based lesion-symptom mapping (VLSM, right panel), comparing the performance of patients with and without a lesion of each voxel, with voxelwise Z statistics corrected for multiple comparisons over the whole brain (threshold: FDR p<0.05). Significant regions are contiguous to the peaks of the corresponding functional areas as identified in normal subjects (red crosshair). Addition of normalized lesions (left panel) for patients with a deficit for words (n = 3; top panel), faces (n = 9; middle panel), and houses (n = 10; bottom panel), minus the lesions of patients with no deficit in the considered category. The regions of maximum overlap are essentially identical to those identified using VLSM.

Results of the analysis of brain imaging are illustrated Figure 8. There was a perfect overlap between the regions showing a statistically significant lesion-symptom link (Figure 8, right panel), and the regions identified by the simple thresholded algebraic combination of lesions (Figure 8, left panel). In all cases, ventral and mesial temporal regions are implicated by visual analysis, strictly left-lateralized for words and right-lateralized for faces and houses, affecting the lingual gyrus and extending to the fusiform gyrus. For words, the maximum overlap of lesions fell in the left fusiform and parahippocampal gyri, contiguous and mesial to the peak of the VWFA. For faces, the pattern was roughly symmetrical to the pattern for words: lesions overlapped maximally in the right fusiform and parahippocampal gyri, contiguous and mesial to the peak of the FFA. For houses, the pattern was similar to the pattern for faces. The overlap of lesions was maximum posterior to the peak of the right PPA, which was nevertheless included in some of the lesions.

We now briefly summarize for each category which patients were considered as impaired in this analysis (a systematic discussion of individual lesions is reported as Text S1).

Three patients had an impaired perception of words (Figure S2): patients #6 (selective deficit on the Array test), #9 and #12 (impairment on the timed reading test). All the other patients with a left-hemispheric lesion (#1 to #15) showed no difficulties with words. Patient #8 had dense macular hemianopia and no word length effect, and his errors on the timed reading test were restricted to the rightmost part of words. His reading impairment likely resulted from hemianopic alexia rather than from pure alexia [19], [23], [24], in agreement with posterior occipital location of his lesion.

Nine patients were identified as having an impaired perception of faces: patients #12, #22, #25 and #27 (impaired on the CMT, Figure S4), #19 and #26 (selective deficit on the Array test), #20 and #21 (selective deficit on the Old/New test), and #31 (selective deficit on the Array test and on the Old/New test). Note that patient #10 (Figure S4) had several deficits, on the Array test for houses and on the CMT with faces and phones, without lesion of the FFA, the PPA or the LOC. We considered that this unexpected pattern likely resulted from simultanagnosia, as further confirmed by his deficit on the Navon test.

Ten patients were identified as having an impaired perception of houses: patients #1, #16, #19, #22, #25, #27 and #31 (impaired on the CMT, Figure S3), #12 and #21 (impaired non-selectively on the Array test), and #29 (selective deficit on the Old/New test).

## Discussion

### Are agnosic deficits really exceptional?

Our first goal was to determine whether agnosic visual deficits are as exceptional as generally reported in series of PCA patients, or whether careful screening would actually reveal a higher incidence of deficit. To this end, we tested a consecutive series of patients with a battery of standardized cognitive tests, plus custom-made tests assessing the perception of important categories of familiar visual objects, including faces, words, houses and tools. Indeed, we identified 15 patients (48%) with an impairment on the standardized visual tests, and as many as 20 patients (65%) with an impairment on the experimental tests. Considering only the CMT, 11 patients (35%) showed a deficit with at least one category (faces, houses or phones). Those figures are clearly higher than in previously reported series of patients, in which the neuropsychological tests that were used are rarely specified with precision [1], [2], [3], [4], [5], [6], [7]. Indeed previous studies report a maximum frequency of 8.5% in isolated superficial PCA infarctions [6]. Naturally, the impairments which we put to light by using sensitive tests were too moderate to induce a substantial handicap, and to be clinically identified as full-fledged visual agnosias.

### Picking out selective deficits: a case of impaired perception of houses

Putting aside case-report studies [16], [19], [24], [59], [60], [61], the selectivity of visual agnosias is generally not specified in large series of patients. In the present study, the selectivity of deficits could be assessed confidently, as the different classes of stimuli were evaluated using carefully matched testing procedures.

To illustrate this point, we briefly discuss the case of patient #1, in whom we identified a specific visual impairment for houses. His score on the CMT with houses was not only pathological compared to controls, but also, according to the stringent RSDT criterion, lower than on the CMT with other stimuli, which were normal. This patient had a haemorrhagic lesion affecting his left parahippocampic gyrus from coordinates y = −47 to y = −73, that is abutting posteriorly the peak of the PPA (see Figure S3).

In a review of neuropsychological case reports, Epstein [62] concludes that lesions of the PPA spare the knowledge of the spatial relationships between locations (large scale mental map), as well as the ability to recognize familiar landmarks (which can presumably be achieved on the basis of the general object-recognition system). Conversely lesions of the PPA would impair the ability to encode the topographical layout of environments, familiar or novel, on the basis of their geometrical features. In contrast, the retrieval of long-term spatial knowledge would depend on the retrosplenial cortex [63]. Patient #1's performance fits with this hypothetical role of the PPA. Indeed, his impairment concerned particularly the second part of the CMT. This test specifically requires subjects to match images of houses across changes in viewpoint and lighting, a task clearly requiring an invariant encoding of the geometrical structure of complex environments.

### Can deficits be predicted from the location of the lesions?

In addition to estimating the actual frequency of agnosic deficits following PCA lesions, our goal was to study anatomical correlations, with the specific expectation that the degree of functional lateralization should have an impact on the occurrence of deficits. We discuss here only the standard case of right-handed subjects, leaving aside the variability of hemispheric dominance in left-handed subjects, an issue which has been mostly studied in the case of language [64]. Note that only one patient (#15) in our study was left-handed. His lesion spared all the specialized visual areas (PPA, FFA, LOC, VWFA), and he had no cognitive deficit.

The group study of lesions showed that lesions associated with a deficit for words, faces or houses overlapped maximally in contiguity to the peaks of the corresponding functional regions as identified in normal subjects. On average, functional regions were therefore only partially lesioned, which may explain why we observed mostly moderate deficits, with no major clinical impact.

According to the overview presented in the introduction, deficits for words are more frequent than deficits for faces, while deficits for locations are the rarest. However, this pattern does not fit with the present figures, as we observed few word processing deficits (3 patients among 15 left ACP strokes, 20%), and a larger and roughly equal number of deficits for houses (2 patients with a left ACP stroke, 13%; 6 patients among 13 right ACP strokes, 46%; 2 patients with a bilateral ACP stroke) and faces (1 patient with a left ACP stroke, 7%; 7 patients with a right PCA stoke, 54%; 1 patient with a bilateral ACP stroke). One reason for this discrepancy may be the sampling of lesions in the present series. Indeed, as shown in Figure 4, lesions affected predominantly the most mesial part of the ventral occipitotemporal cortex (i.e. the calcarine artery territory, which is the most frequently involved in PCA strokes [6]). The PPA (parahippocampal gyrus) is more mesial than the FFA (fusiform gyrus), which is in turn more mesial than the VWFA (lateral occipitotemporal sulcus). This bias may explain the relatively high frequency of deficits for houses and faces as compared to words.

Moreover, most of the category-selective regions which we consider are actually bilateral, with a variable degree of activation asymmetry: in most subjects, the VWFA is strongly left-lateralized [65], the FFA moderately right-lateralized (see [66] for a review), and the PPA roughly symmetrical [13]. If we assume that the more symmetrical a region, the better the intact hemisphere can compensate for the effects of a unilateral lesion, then pure alexia should be expected to be more frequent than prosopagnosia, which in turn would be more frequent than place agnosia, in cases of lesions of the VWFA, FFA and PPA, respectively. In other terms, anatomical-clinical correlations should be looser with more bilateral functions. The occurrence of a deficit would depend not only on the precise intra-hemispheric topography of the lesion, but also on the premorbid functional asymmetry of the relevant region. As the VWFA is strongly left-lateralized in most subjects, lesions of this structure should be good predictors of the occurrence of pure alexia [19], [25]. Conversely, prosopagnosia or place agnosia should occur only in the subset patients who had a marked premorbid asymmetry of the FFA or PPA. We now discuss how each category of stimulus fit this general schema.

#### Words

Printed words may be the only category for which previous studies provide both information on the distribution of functional lateralization in normal subjects, and detailed anatomical-clinical correlations in groups of patients. All group studies contrasting activations of the VWFA by words relative to other complex visual stimuli find exclusively or predominantly left-hemispheric activations [67], [68]. Thus all 16 right-handed subjects reported by Cohen et al. [65] had left-hemispheric activations of the VWFA by words relative to checkerboards. Among them, 2 (12%) also had smaller activations in the symmetrical right-hemispheric region. Using a similar contrast, Cai et al. [64] found as many as 5 subjects out of 16 with right-hemispheric activations and 2 with bilateral activations. However, as 9 out of the 16 subjects were left-handers, this distribution is obviously not representative of the right-handers population. In agreement with the strong left lateralization present in a large majority of right-handed subjects, tight correlations exist between lesions of the VWFA and the occurrence of pure alexia [19], [25].

In our study, out of the 5 patients whose lesions overlapped at least partially with the VWFA region of interest, 3 patients (#6, #9 and #12) had a word processing impairment (60%). The other two (#1 and #5) had no deficit with words. Their lesions were just in contact with the VWFA, but spared most of the region of interest. Conversely, no lesion sparing the VWFA induced deficit with words. So, anatomical-clinical correlations for words are strong and coherent with previous reports, particularly regarding the left-hemispheric lateralization.

#### Faces

Imaging studies provide coherent information about the normal distribution of lateralization of activations during face recognition (see Dien [66] for a review). The FFA, as identified by contrasting faces against objects, is generally right-lateralized. Out of seven studies using an explicit statistical test to evaluate the FFA lateralization, four reported right-hemispheric lateralization [69], [70], [71], [72], two reported a non-significant trend towards right-hemispheric lateralization [8], [73], and two reported symmetrical activations [74], [75]. In addition, despite exceptions observed in left-handed patients [76], prosopagnosia systematically results from bilateral [77] or unilateral right occipitotemporal lesion [16].

In our study, right lesions tend to be associated with a higher risk of deficit: all 6 lesions (patients #21, #22, #25, #26, #27 and #31) affecting the right FFA induced a deficit, while only one lesion (patient #12) affecting the left FFA (17%) induced a deficit. Those results are congruent with previous reports and with the hypothesis of a predominant premorbid asymmetry of the FFA, more often toward the right-hemisphere. However, anatomical-clinical correlations for faces are less strong than for words: one patient (#29) with a bilateral lesion including the two FFA (sparing only the right OFA) had no deficit with faces, and 3 patients (#10, #19 and #20) without lesion of the FFA (patient #19 with a lesion of the right OFA) had difficulties with faces.

#### Houses

The normal pattern of lateralization is much less studied for the PPA than for the VWFA or the FFA. Activations of the PPA by scenes relative to faces or objects are generally bilateral [13], [56], with an occasional right-hemispheric predominance [13]. Lesions studies with topographical disorientation, loosely defined as an impairment of spatial orientation and navigation, concern over 200 patients [78]. In a subset of patients, the deficit results from damage to the PPA, with a predominant right hemispheric involvement [79]. In a study of 127 subjects with focal lesions [21], all the 9 patients with damage to the right PPA suffered from topographical disorientation, and 4 out of 7subjects with damage to the left PPA were impaired. Lesions affecting scene and building recognition, i.e. place agnosia per se, always involve the right PPA, but are very rare, amounting to less than 30 cases [62], [79], [80], [81], [82], [83], [84], [85].

In our study, the category of houses was frequently impaired: among the 6 lesions affecting only the left PPA, 2 induced a deficit (33%, patients #1 and #12), while among the 11 lesions affecting only the right PPA, 6 induced a deficit (55%, patients #19, #21, #22, #25, #27 and #31). Those results are thus in agreement with previous reports, showing a slight right hemispheric predominance.

In summary, while a lesion of the VWFA is a strong predictor of reading impairment, probably because of the marked left-hemispheric lateralization of the VWFA anatomical-clinical correlations are weaker for lesions of the FFA and the PPA as predictors of deficits for faces or places, as those structures are generally bilateral, with substantial individual variability. A relative preponderance of right-hemispheric lesion seem however to be typical, particularly as concerns the perception of faces.

In conclusion, this study allowed us to demonstrate that high-level visual deficits are more frequent than previously reported following PCA strokes affecting the ventral visual cortex, provided sufficiently sensitive tests are used. The strength of anatomical-clinical correlations differs across categories of visual objects, depending on the pattern of lateralization of each category-selective visual area. The VWFA is markedly left lateralized in most subjects, and lesions are therefore highly predictive of reading impairments. In contrast, the FFA and PPA are actually bilateral with an average right-hemispheric bias, explaining the relative rarity of agnosias for faces and places, and the lower predictive value of focal lesion on the occurrence of a deficit.

## Supporting Information

Figure S1 Review image combining frontal views of the 6 target items for houses (left panel), and for phones (right panel).
(TIF)

Figure S2 Reconstruction of the lesions of patients #6 (in blue), 8 (in violet), 9 (in green) and 12 (in yellow) in Talairach space, compared with the average normal location of the VWFA (red dot). Slices are TC z = −12 and y = −58.
(TIF)

Figure S3 Reconstruction of the lesions of patients #1 (in blue), 16 (in orange), 19 (in violet), 22 (in green), 25 (in yellow), 27 (in cyan) and 31 (in brown) in Talairach space, compared with the average normal location of the left and right PPA (red dots). Slices are TC z = −14 [#16, z = 2] and x = 29 [#1, x = −27].
(TIF)

Figure S4 Reconstruction of the lesions of patients #10 (in blue), 12 (in yellow), 22 (in green), 25 (in violet) and 27 (in cyan) in Talairach space, compared with the average normal location of the left and right FFA (red dots). Slices are TC z = −10 and y = −58.
(TIF)

Figure S5 Reconstruction of the lesions of patients #10 (in blue), 24 (in green), 25 (in yellow), 26 (in violet) and 31 (in cyan) in Talairach space, compared with the average normal location of the left and right LOC (red dots). Slices are TC z = −6 and y = −76.
(TIF)

Table S1 Neuropsychological findings in 31 patients.
(DOC)

Table S2 Experimental study in 31 patients (and 41controls).
(DOC)

Table S3 Reading test in 31 patients (and 41controls).
(DOC)

Table S4 Results for CMT in 31 patients (and 41 controls).
(DOC)

Text S1 Supplemental data.
(DOC)

## References

[1] Yamamoto Y, Georgiadis AL, Chang HM, Caplan LR (1999). Posterior cerebral artery territory infarcts in the New England Medical Center Posterior Circulation Registry.. Arch Neurol.

[2] Pessin MS, Lathi ES, Cohen MB, Kwan ES, Hedges TR (1987). Clinical features and mechanism of occipital infarction.. Ann Neurol.

[3] Servan J, Catala M, Rancurel G (1992). Posterior cerebral artery infarction: a study of 76 cases [abstract].. Cerebrovasc Dis.

[4] Milandre L, Brosset C, Botti G, Khalil R (1994). [A study of 82 cerebral infarctions in the area of posterior cerebral arteries].. Rev Neurol (Paris).

[5] Brandt T, Thie A, Caplan LR, Hacke W (1995). [Infarcts in the brain areas supplied by the posterior cerebral artery. Clinical aspects, pathogenesis and prognosis].. Nervenarzt.

[6] Cals N, Devuyst G, Afsar N, Karapanayiotides T, Bogousslavsky J (2002). Pure superficial posterior cerebral artery territory infarction in The Lausanne Stroke Registry.. J Neurol.

[7] Kumral E, Bayulkem G, Atac C, Alper Y (2004). Spectrum of superficial posterior cerebral artery territory infarcts.. Eur J Neurol.

[8] Puce A, Allison T, Gore JC, McCarthy G (1995). Face-sensitive regions in human extrastriate cortex studied by functional MRI.. J Neurophysiol.

[9] Kanwisher N, McDermott J, Chun M (1997). The fusiform face area: a module in human extrastriate cortex specialized for face perception.. J Neurosci.

[10] Gauthier I, Tarr MJ, Moylan J, Skudlarski P, Gore JC (2000). The fusiform “face area” is part of a network that processes faces at the individual level.. J Cogn Neurosci.

[11] Cohen L, Dehaene S, Naccache L, Lehericy S, Dehaene-Lambertz G (2000). The visual word form area: spatial and temporal characterization of an initial stage of reading in normal subjects and posterior split-brain patients.. Brain.

[12] Downing PE JY, Shuman M, Kanwisher N (2001). A cortical area selective for visual processing of the human body.. Science.

[13] Epstein R, Kanwisher N (1998). A cortical representation of the local visual environment.. Nature.

[14] Malach R, Reppas JB, Benson RR, Kwong KK, Jiang H (1995). Object-related activity revealed by functional magnetic resonance imaging in human occipital cortex.. Proc Natl Acad Sci U S A.

[15] Downing PE, Chan AW, Peelen MV, Dodds CM, Kanwisher N (2006). Domain specificity in visual cortex.. Cereb Cortex.

[16] Farah M (2004).

[17] Rossion B, Caldara R, Seghier M, Schuller AM, Lazeyras F (2003). A network of occipito-temporal face-sensitive areas besides the right middle fusiform gyrus is necessary for normal face processing.. Brain.

[18] Busigny T, Graf M, Mayer E, Rossion B (2010). Acquired prosopagnosia as a face-specific disorder: ruling out the general visual similarity account.. Neuropsychologia.

[19] Cohen L, Martinaud O, Lemer C, Lehericy S, Samson Y (2003). Visual word recognition in the left and right hemispheres: anatomical and functional correlates of peripheral alexias.. Cereb Cortex.

[20] Gaillard R, Naccache L, Pinel P, Clemenceau S, Volle E (2006). Direct intracranial, FMRI, and lesion evidence for the causal role of left inferotemporal cortex in reading.. Neuron.

[21] Barrash J, Damasio H, Adolphs R, Tranel D (2000). The neuroanatomical correlates of route learning impairment.. Neuropsychologia.

[22] Peelen MV, Downing PE (2007). The neural basis of visual body perception.. Nat Rev Neurosci.

[23] Leff AP, Crewes H, Plant GT, Scott SK, Kennard C (2001). The functional anatomy of single-word reading in patients with hemianopic and pure alexia.. Brain.

[24] Leff AP, Spitsyna G, Plant GT, Wise RJ (2006). Structural anatomy of pure and hemianopic alexia.. J Neurol Neurosurg Psychiatry.

[25] Pflugshaupt T, Gutbrod K, Wurtz P, von Wartburg R, Nyffeler T (2009). About the role of visual field defects in pure alexia.. Brain.

[26] Henry C, Gaillard R, Volle E, Chiras J, Ferrieux S (2005). Brain activations during letter-by-letter reading: a follow-up study.. Neuropsychologia.

[27] Duchaine BC, Parker H, Nakayama K (2003). Normal recognition of emotion in a prosopagnosic.. Perception.

[28] Duchaine B, Nakayama K (2005). Dissociations of face and object recognition in developmental prosopagnosia.. J Cogn Neurosci.

[29] Warrington E (1984). Recognition memory test.

[30] Benton AL, Sivan AB, Hamsher KD, Varney NR, Spreen O (1983). Contribution to neuropsychological assessment.

[31] Duchaine BC, Weidenfeld A (2003). An evaluation of two commonly used tests of unfamiliar face recognition.. Neuropsychologia.

[32] Tatu L, Moulin T, Bogousslavsky J, Duvernoy H (1998). Arterial territories of the human brain: cerebral hemispheres.. Neurology.

[33] Oldfield RC (1971). The assessment and analysis of handedness: the Edinburgh inventory.. Neuropsychologia.

[34] Folstein M, Folstein S, Mc Hugh P (1975). Mini-mental state. A practical method for grading the cognitive state of patients for the clinician.. JPsychiatr Res.

[35] Deloche G, Hannequin D (1997). DO80.

[36] De Renzi E, Faglioni P (1978). Normative data and screening power of a shortened version of the Token Test.. Cortex.

[37] Wechsler D (1997). Wechsler Adult Intelligence Scale Third edition.

[38] Van der Linden M, Coyette F, Poitrenaud J, Kalafat M, Calicis F, Van der Linden M (2004). L'épreuve de rappel libre/rappel indicé à 16 items (RL/RI-16).. L'évaluation des troubles de la mémoire.

[39] Baddeley A, Emslie H, Nimmo-Smith I (1994). The Doors and People Test: a test of visual and verbal recall and recognition..

[40] Sivan AB (1992). Benton Visual Retention Test Fifth Edition.

[41] Agniel A, Joanette Y, Doyon B, Duchein C (1992). Protocole Montreal-Toulouse. Evaluation des gnosies visuelles et auditives.

[42] Navon D (1977). Forest before the trees: the precedence of global features in visual perception.. Cogn Psychol.

[43] Riddoch MJ, Humphreys GW (1993). BORB: Birmingham Object Recognition Battery.

[44] Benton AL, Varney NR, Hamsher KD (1978). Visuospatial judgment. A clinical test.. Arch Neurol.

[45] Garrido L, Duchaine B, Nakayama K (2008). Face detection in normal and prosopagnosic individuals.. J Neuropsychol.

[46] Vinckier F, Dehaene S, Jobert A, Dubus JP, Sigman M (2007). Hierarchical coding of letter strings in the ventral stream: dissecting the inner organization of the visual word-form system.. Neuron.

[47] Duchaine B, Nakayama K (2006). The Cambridge Face Memory Test: Results for neurologically intact individuals and an investigation of its validity using inverted face stimuli and prosopagnosic participants.. Neuropsychologia.

[48] Pourtois G, Schwartz S, Seghier ML, Lazeyras F, Vuilleumier P (2005). Portraits or people? Distinct representations of face identity in the human visual cortex.. J Cogn Neurosci.

[49] Epstein R, Graham KS, Downing PE (2003). Viewpoint-specific scene representations in human parahippocampal cortex.. Neuron.

[50] Grill-Spector K (2003). The neural basis of object perception.. Curr Opin Neurobiol.

[51] Duchaine BC, Nieminen-von Wendt T, New J, Kulomaki T (2003). Dissociations of visual recognition in a developmental agnosic: evidence for separate developmental processes.. Neurocase.

[52] Golarai G, Ghahremani DG, Whitfield-Gabrieli S, Reiss A, Eberhardt JL (2007). Differential development of high-level visual cortex correlates with category-specific recognition memory.. Nat Neurosci.

[53] Crawford JR, Garthwaite PH (2005). Evaluation of criteria for classical dissociations in single-case studies by Monte Carlo simulation.. Neuropsychology.

[54] Peelen MV, Downing PE (2005). Selectivity for the human body in the fusiform gyrus.. J Neurophysiol.

[55] Dehaene S, Cohen L (2007). Cultural Recycling of Cortical Maps.. Neuron.

[56] Park S, Chun MM (2009). Different roles of the parahippocampal place area (PPA) and retrosplenial cortex (RSC) in panoramic scene perception.. Neuroimage.

[57] Downing PE, Wigett AJ, Peelen MV (2007). Functional Magnetic Resonance Imaging Investigation of Overlapping Lateral Occipitotemporal Activations Using Multi-Voxel Pattern Analysis.. J Neurosci.

[58] Bates E, Wilson SM, Saygin AP, Dick F, Sereno MI (2003). Voxel-based lesion-symptom mapping.. Nat Neurosci.

[59] De Renzi E (1986). Prosopagnosia in two patients with CT scan evidence of damage confined to the right hemisphere.. Neuropsychologia.

[60] Damasio AR, Damasio H (1983). The anatomic basis of pure alexia.. Neurology.

[61] Binder JR, Mohr JP (1992). The topography of callosal reading pathways. A case-control analysis.. Brain.

[62] Epstein RA (2008). Parahippocampal and retrosplenial contributions to human spatial navigation.. Trends Cogn Sci.

[63] Epstein RA, Parker WE, Feiler AM (2007). Where am I now? Distinct roles for parahippocampal and retrosplenial cortices in place recognition.. J Neurosci.

[64] Cai Q, Paulignan Y, Brysbaert M, Ibarrola D, Nazir TA (2010). The left ventral occipito-temporal response to words depends on language lateralization but not on visual familiarity.. Cereb Cortex.

[65] Cohen L, Lehericy S, Chochon F, Lemer C, Rivaud S (2002). Language-specific tuning of visual cortex? Functional properties of the Visual Word Form Area.. Brain.

[66] Dien J (2009). A tale of two recognition systems: implications of the fusiform face area and the visual word form area for lateralized object recognition models.. Neuropsychologia.

[67] Cohen L, Jobert A, Le Bihan D, Dehaene S (2004). Distinct unimodal and multimodal regions for word processing in the left temporal cortex.. Neuroimage.

[68] Jobard G, Crivello F, Tzourio-Mazoyer N (2003). Evaluation of the dual route theory of reading: a metanalysis of 35 neuroimaging studies.. Neuroimage.

[69] Gauthier I, Tarr MJ, Anderson AW, Skudlarski P, Gore JC (1999). Activation of the middle fusiform ‘face area’ increases with expertise in recognizing novel objects.. Nat Neurosci.

[70] Haxby JV, Ungerleider LG, Clark VP, Schouten JL, Hoffman EA (1999). The effect of face inversion on activity in human neural systems for face and object perception.. Neuron.

[71] Rhodes G, Byatt G, Michie PT, Puce A (2004). Is the fusiform face area specialized for faces, individuation, or expert individuation?. J Cogn Neurosci.

[72] Ishai A, Schmidt CF, Boesiger P (2005). Face perception is mediated by a distributed cortical network.. Brain Res Bull.

[73] Gilaie-Dotan S, Malach R (2007). Sub-exemplar Shape Tuning in Human Face-Related Areas.. Cereb Cortex.

[74] Haxby JV, Grady CL, Horwitz B, Ungerleider LG, Mishkin M (1991). Dissociation of object and spatial visual processing pathways in human extrastriate cortex.. Proc Natl Acad Sci U S A.

[75] Ishai A, Haxby JV, Ungerleider LG (2002). Visual imagery of famous faces: effects of memory and attention revealed by fMRI.. Neuroimage.

[76] Barton JJ (2008). Prosopagnosia associated with a left occipitotemporal lesion.. Neuropsychologia.

[77] Damasio AR, Damasio H, Van Hoesen GW (1982). Prosopagnosia: anatomic basis and behavioral mechanisms.. Neurology.

[78] Barrash J (1998). A historical review of topographical disorientation and its neuroanatomical correlates.. J Clin Exp Neuropsychol.

[79] Aguirre GK, D'Esposito M (1999). Topographical disorientation: a synthesis and taxonomy.. Brain.

[80] Mendez MF, Cherrier MM (2003). Agnosia for scenes in topographagnosia.. Neuropsychologia.

[81] Epstein R, Deyoe EA, Press DZ, Rosen AC, Kanwisher N (2001). Neuropsychological evidence for a topographical learning mechanism in parahippocampal cortex.. Cogn Neuropsychol.

[82] Habib M, Sirigu A (1987). Pure topographical disorientation: a definition and anatomical basis.. Cortex.

[83] Hecaen H, Tzortzis C, Rondot P (1980). Loss of topographic memory with learning deficits.. Cortex.

[84] Takahashi N, Kawamura M (2002). Pure topographical disorientation–the anatomical basis of landmark agnosia.. Cortex.

[85] Sewards TV (2011). Neural structures and mechanisms involved in scene recognition: A review and interpretation.. Neuropsychologia.

