# Supplementary figures and images for: Visual Agnosia and Posterior Cerebral Artery Infarcts: An Anatomical-Clinical Study
Source: PLoS One. 2012 Jan 20;7(1):e30433. doi: 10.1371/journal.pone.0030433 (PMC3262828; doi:10.1371/journal.pone.0030433)

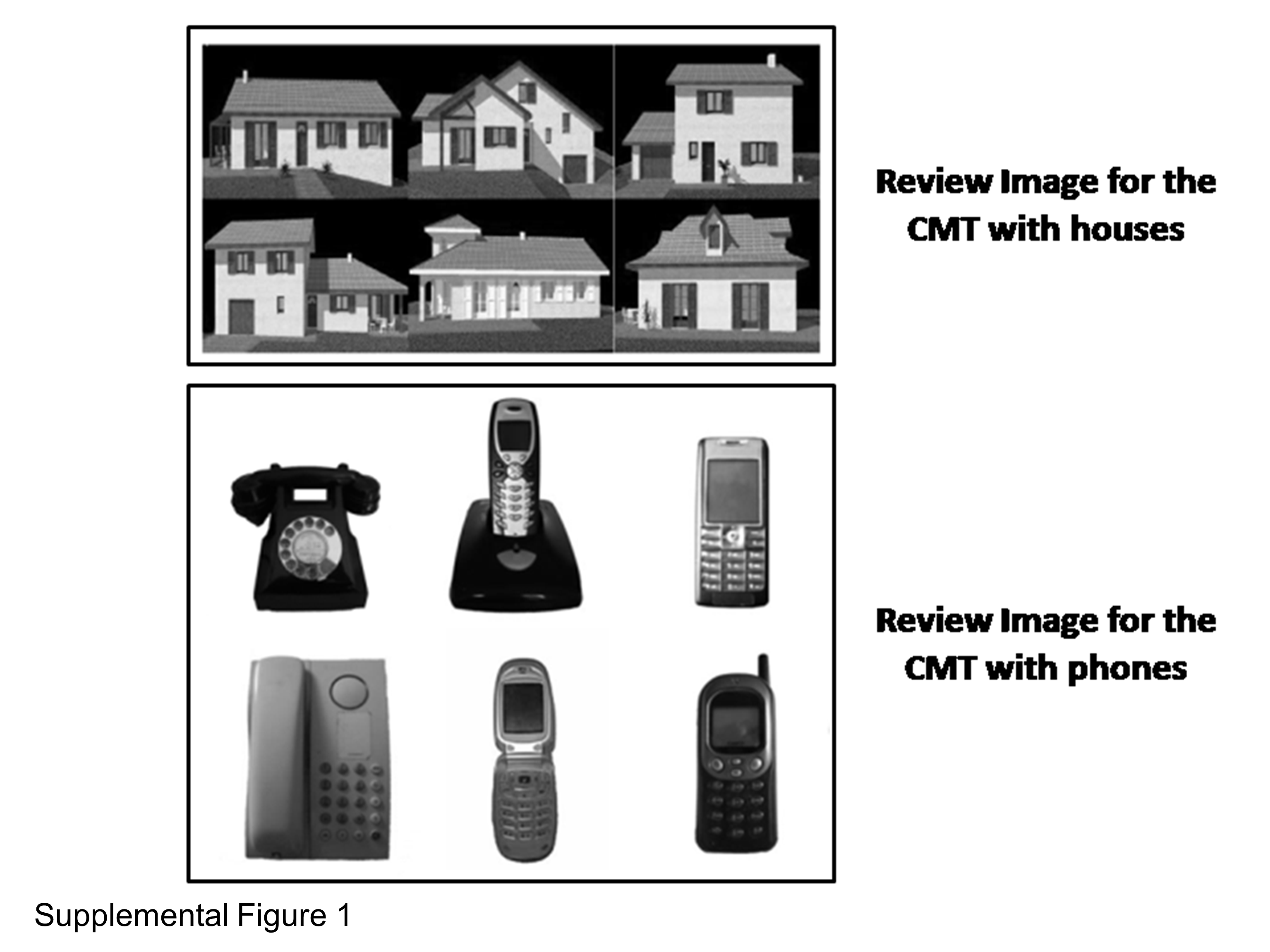

Supplement: Figure S1 — Review image combining frontal views of the 6 target items for houses (left panel), and for phones (right panel). (TIF) [file pone.0030433.s001.tif]

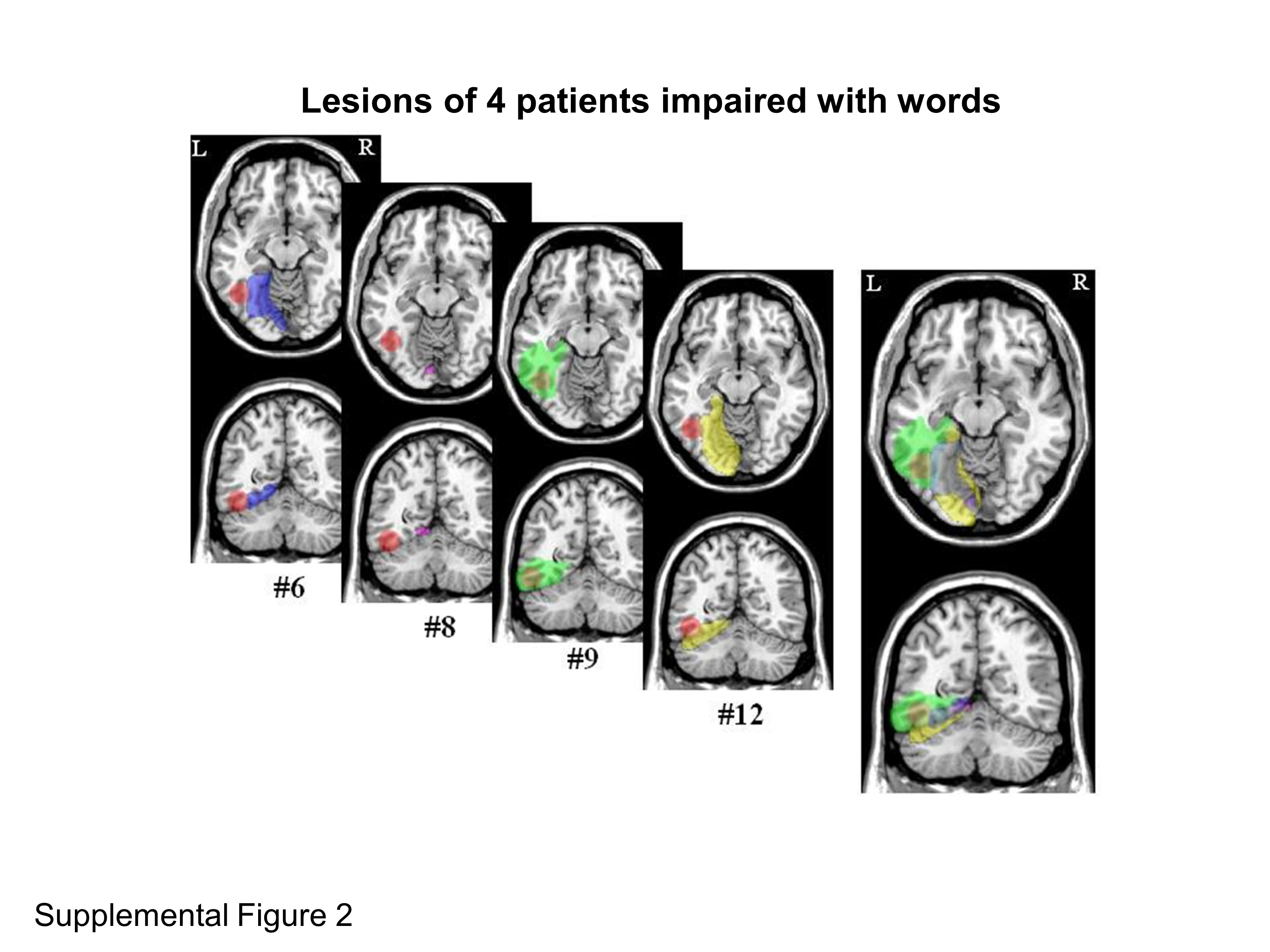

Supplement: Figure S2 — Reconstruction of the lesions of patients #6 (in blue), 8 (in violet), 9 (in green) and 12 (in yellow) in Talairach space, compared with the average normal location of the VWFA (red dot). Slices are TC z = −12 and y = −58. (TIF) [file pone.0030433.s002.tif]

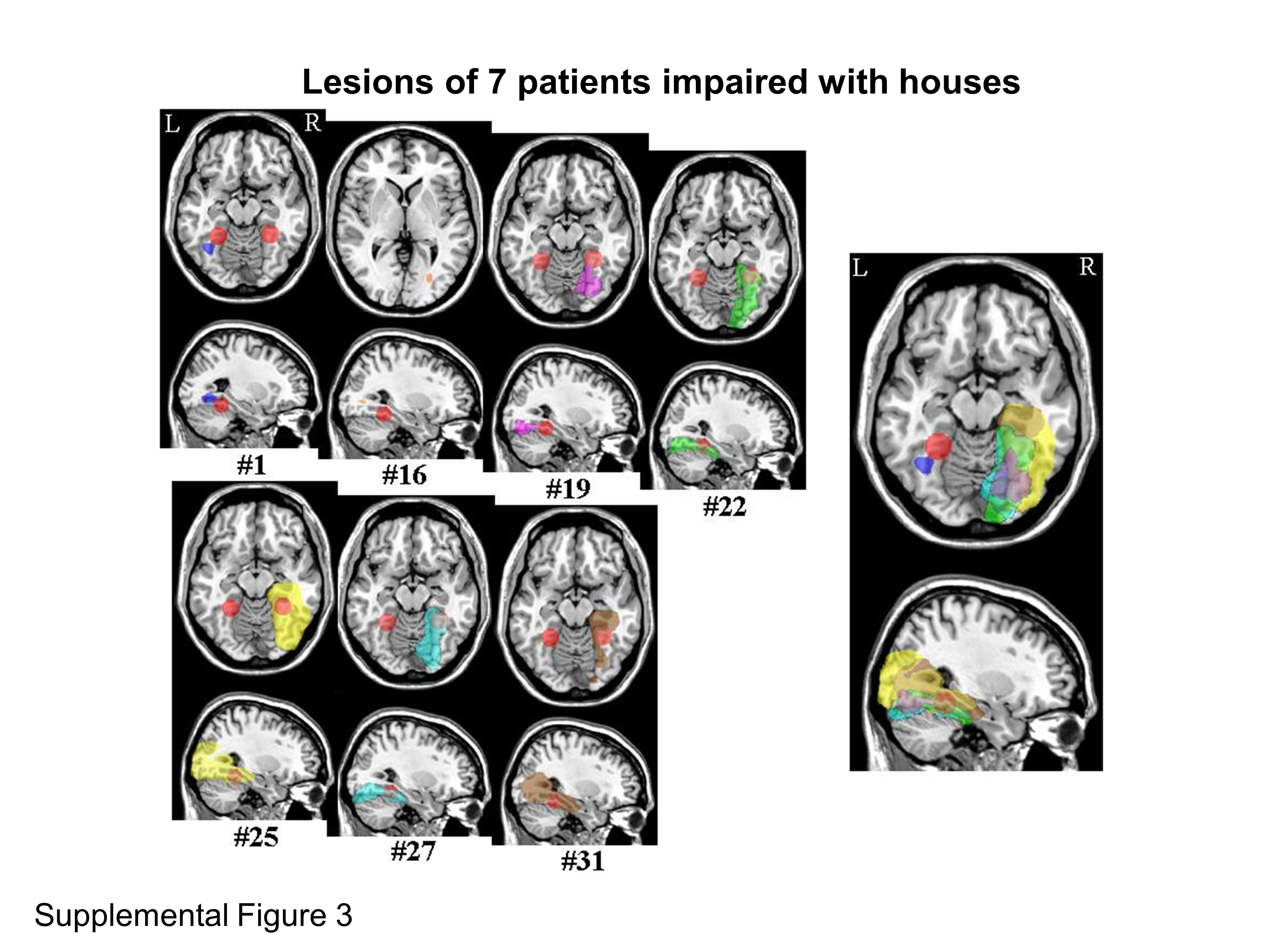

Supplement: Figure S3 — Reconstruction of the lesions of patients #1 (in blue), 16 (in orange), 19 (in violet), 22 (in green), 25 (in yellow), 27 (in cyan) and 31 (in brown) in Talairach space, compared with the average normal location of the left and right PPA (red dots). Slices are TC z = −14 [#16, z = 2] and x = 29 [#1, x = −27]. (TIF) [file pone.0030433.s003.tif]

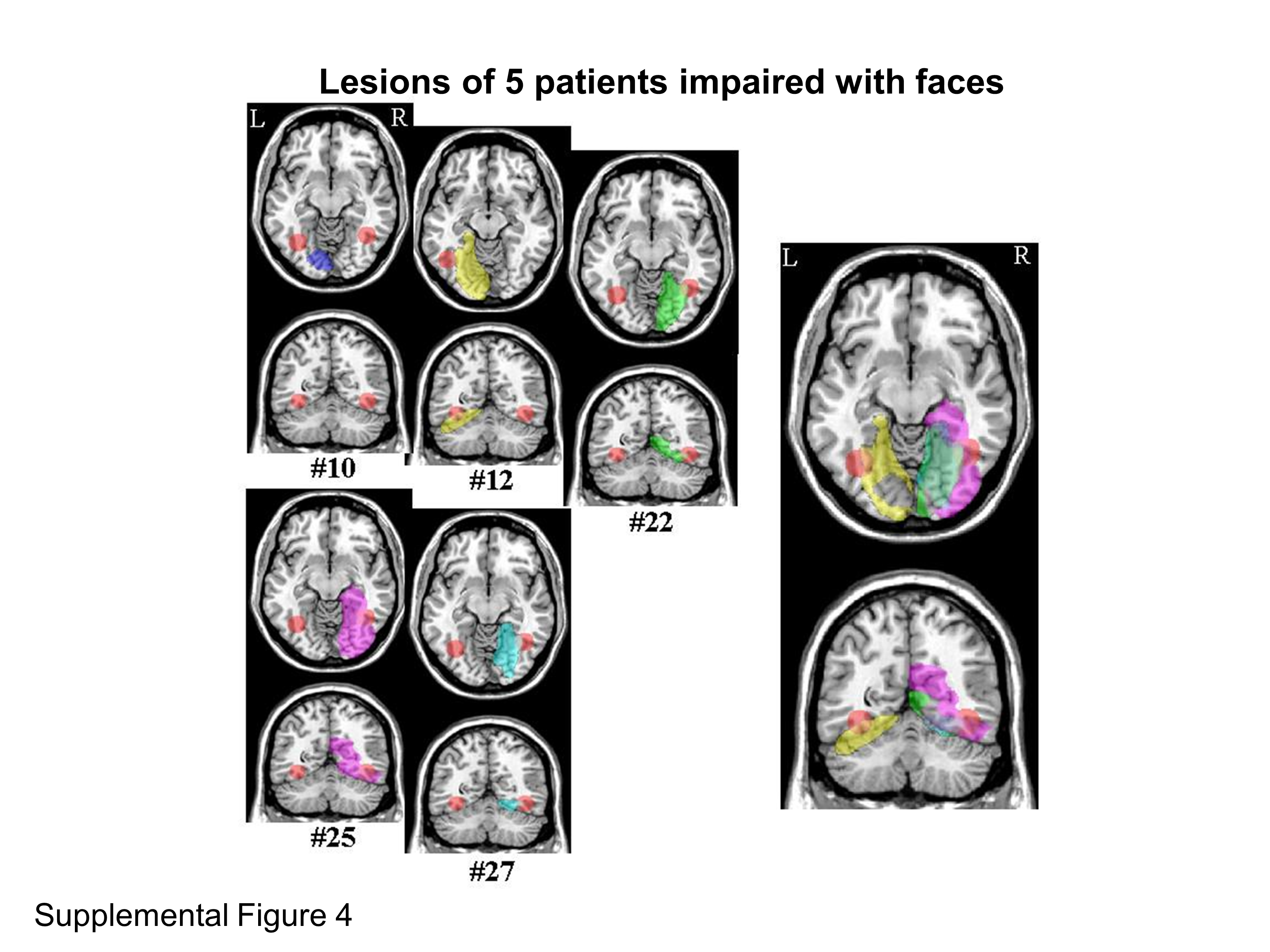

Supplement: Figure S4 — Reconstruction of the lesions of patients #10 (in blue), 12 (in yellow), 22 (in green), 25 (in violet) and 27 (in cyan) in Talairach space, compared with the average normal location of the left and right FFA (red dots). Slices are TC z = −10 and y = −58. (TIF) [file pone.0030433.s004.tif]

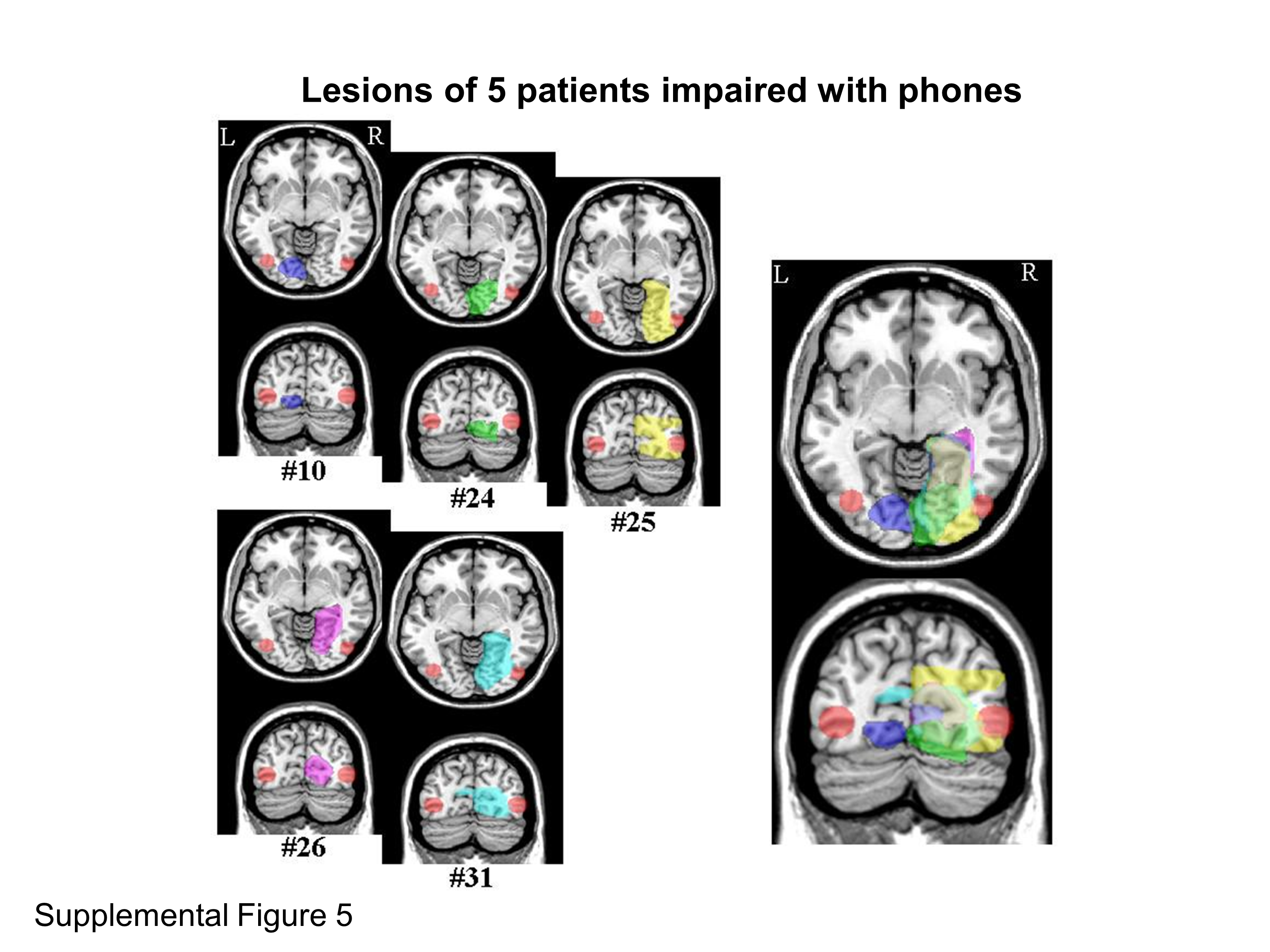

Supplement: Figure S5 — Reconstruction of the lesions of patients #10 (in blue), 24 (in green), 25 (in yellow), 26 (in violet) and 31 (in cyan) in Talairach space, compared with the average normal location of the left and right LOC (red dots). Slices are TC z = −6 and y = −76. (TIF) [file pone.0030433.s005.tif]
