# Supplementary material for: Visual Agnosia and Posterior Cerebral Artery Infarcts: An Anatomical-Clinical Study
Source: PLoS One. 2012 Jan 20;7(1):e30433. doi: 10.1371/journal.pone.0030433 (PMC3262828; doi:10.1371/journal.pone.0030433)
Supplement: Table S1 — Neuropsychological findings in 31 patients. (DOC) [file pone.0030433.s006.doc]

***Table 1*** *Neuropsychological findings in 31 patients*

|  | **Left (n=15)** | | | | **Right (n=13)** | | | | **Bilateral (n=3)** | | | |
| --- | --- | --- | --- | --- | --- | --- | --- | --- | --- | --- | --- | --- |
|  | Deficit | | Performance | | Deficit | | Performance | | Deficit | | Performance | |
|  | n | % | Mean | Range | n | % | Mean | Range | n | % | Mean | Range |
| Global Efficiency | **3** | **9.7** |  |  | **0** | **0** |  |  | **0** | **0** |  |  |
| MMS | 3 | 9.7 | 26.8 | 24-30 | 0 | 0 | 28 | 25-30 | 0 | 0 | 27 | 26-28 |
| Language | **2** | **6.4** |  |  | **0** | **0** |  |  | **0** | **0** |  |  |
| DO80 | 2 | 6.4 | 77.6 | 69-80 | 0 | 0 | 77.9 | 73-80 | 0 | 0 | 78.3 | 77-80 |
| Token test | 0 | 0 | 33.5 | 31-36 | 0 | 0 | 33.5 | 31,5-36 | 0 | 0 | 33.3 | 30-36 |
| Working Memory | **0** | **0** |  |  | **2** | **6.4** |  |  | **0** | **0** |  |  |
| Digit span (forward/backward) | 0 | 0 | 5.5/3.9 | 3-8/3-7 | 1 | 3.2 | 5.6/4.1 | 4-7/3-6 | 0 | 0 | 6.3/4.3 | 6-7/4-5 |
| Spatial span (forward/backward) | 0 | 0 | 5.7/5.1 | 5-7/3-7 | 1 | 3.2 | 5.6/5.4 | 4-10/4-7 | 0 | 0 | 5.7/5.3 | 4-7/4-7 |

MMS Mini Mental State; DO80 oral confrontation naming test; GBVLT Grober and Buschke Verbal Learning test; PEGV 'Protocole Montréal-Toulouse d'évaluation des gnosies visuelles'; BORB Birmingham Object Recognition Battery; JLOT Benton judgment of line orientation test; n = number of patients with deficit; % = percentage of patients with deficit.

|  | **Left (n=15)** | | | | **Right (n=13)** | | | | **Bilateral (n=3)** | | | |
| --- | --- | --- | --- | --- | --- | --- | --- | --- | --- | --- | --- | --- |
|  | Deficit | | Performance | | Deficit | | Performance | | Deficit | | Performance | |
|  | n | % | Mean | Range | n | % | Mean | Range | n | % | Mean | Range |
| Memory |  |  |  |  |  |  |  |  |  |  |  |  |
| *Verbal* memory (GBVLT) | **5** | **16.1** |  |  | **5** | **16.1** |  |  | **1** | **3.2** |  |  |
| Sum of 3 free recall | 3 | 9.7 | 26.9 | 19-39 | 3 | 9.7 | 29.7 | 19-44 | 0 | 0 | 31.3 | 27-39 |
| Total recall 3 | 3 | 9.7 | 14.6 | 12-16 | 2 | 6.4 | 15.3 | 12-16 | 1 | 3.2 | 15.3 | 14-16 |
| Differed free recall | 3 | 9.7 | 10.7 | 9-16 | 3 | 9.7 | 11 | 7-16 | 0 | 0 | 12.7 | 10-14 |
| Differed total recall | 3 | 9.7 | 14.6 | 14-16 | 1 | 3.2 | 15.3 | 11-16 | 1 | 3.2 | 15.3 | 14-16 |
| Recognition | 2 | 6.4 | 14.8 | 15-16 | 2 | 6.4 | 15.5 | 13-16 | 0 | 0 | 15.7 | 15-16 |

MMS Mini Mental State; DO80 oral confrontation naming test; GBVLT Grober and Buschke Verbal Learning test; PEGV 'Protocole Montréal-Toulouse d'évaluation des gnosies visuelles'; BORB Birmingham Object Recognition Battery; JLOT Benton judgment of line orientation test; n = number of patients with deficit; % = percentage of patients with deficit.

|  | **Left (n=15)** | | | | **Right (n=13)** | | | | **Bilateral (n=3)** | | | |
| --- | --- | --- | --- | --- | --- | --- | --- | --- | --- | --- | --- | --- |
|  | Deficit | | Performance | | Deficit | | Performance | | Deficit | | Performance | |
|  | n | % | Mean | Range | n | % | Mean | Range | n | % | Mean | Range |
| *Visual Memory* | **5** | **16.1** |  |  | **6** | **19.4** |  |  | **0** | **0** |  |  |
| Doors test part A | 3 | 9.7 | 10.2 | 6-12 | 3 | 9.7 | 8.5 | 3-12 | 0 | 0 | 9.3 | 9-11 |
| Benton recognition | 5 | 16.1 | 11.3 | 7-15 | 4 | 12.9 | 11.1 | 8-15 | 0 | 0 | 12.3 | 11-13 |
| **Visual Perceptual Abilities** | **6** | **19.4** |  |  | **6** | **19.4** |  |  | **3** | **9.7** |  |  |
| PEGV | 0 | 0 | 11.1 | 9-12 | 2 | 6.4 | 10.5 | 7-12 | 1 | 3.2 | 9 | 5-12 |
| BORB | 0 | 0 | 24.1 | 22-25 | 0 | 0 | 23.7 | 22-26 | 0 | 0 | 24.3 | 24-25 |
| JLOT | 5 | 16.1 | 19.2 | 9-27 | 5 | 16.1 | 20.5 | 11-28 | 3 | 9.7 | 13.7 | 11-17 |
| Navon test | 2 | 6.4 | 184.3 | 152-192 | 3 | 9.7 | 183.5 | 163-192 | 1 | 3.2 | 181.7 | 163-191 |

MMS Mini Mental State; DO80 oral confrontation naming test; GBVLT Grober and Buschke Verbal Learning test; PEGV 'Protocole Montréal-Toulouse d'évaluation des gnosies visuelles'; BORB Birmingham Object Recognition Battery; JLOT Benton judgment of line orientation test; n = number of patients with deficit; % = percentage of patients with deficit.
