# Supplementary material for: Visual Agnosia and Posterior Cerebral Artery Infarcts: An Anatomical-Clinical Study
Source: PLoS One. 2012 Jan 20;7(1):e30433. doi: 10.1371/journal.pone.0030433 (PMC3262828; doi:10.1371/journal.pone.0030433)
Supplement: Table S2 — Experimental study in 31 patients (and 41controls). (DOC) [file pone.0030433.s007.doc]

***Table 2*** Experimental study in 31 patients (and 41 controls)

| **Stroke** | N° | **ArrayF** | **ArrayH** | **ArrayP** | **ArrayW** | **CMTFt** | **CMTHt** | **CMTPt** | **ONface** | **ONcar** | **ONflower** | **ONgun** | **ONhorse** | **ONhouse** | **ONglasses** | **ONtool** | **ONscene** |
| --- | --- | --- | --- | --- | --- | --- | --- | --- | --- | --- | --- | --- | --- | --- | --- | --- | --- |
| Left (n=15) | 1 | 84 | 58 | 60 | 62 | 82 | **61*** | 83 | 2.78 | 1.49 | 1.30 | .91 | 1.68 | 4.41 | 1.02 | 1.45 | 2.51 |
|  | 2 | 88 | 62 | **52*** | 68 | 56 | 78 | 82 | .84 | 1.37 | 1.95 | 2.32 | 2.32 | 3.48 | 1.60 | 1.37 | 2.32 |
|  | 3 | 88 | 94 | 86 | 70 | 94 | 86 | 85 | 1.57 | 5.15 | 1.37 | 1.95 | 2.32 | 2.76 | 2.78 | 5.15 | 5.15 |
|  | 4 | 94 | 80 | 90 | 78 | 88 | 90 | 82 | 1.95 | 3.48 | 2.32 | 2.32 | 1.95 | 1.64 | 1.37 | 2.78 | 2.32 |
|  | 5 | 82 | 68 | 68 | 68 | 68 | 85 | 79 | 1.50 | .65 | 1.11 | 1.37 | 1.95 | 5.15 | .21 | 5.15 | 2.78 |
|  | 6 | 78 | 74 | 68 | **60*** | 79 | 89 | 75 | 2.37 | 1.37 | 2.32 | 1.95 | 2.78 | 2.78 | 1.64 | 3.15 | 3.48 |
|  | 7 | 86 | 74 | 82 | 84 | 76 | 94 | 82 | 2.61 | 5.15 | 2.32 | 5.15 | 2.32 | 3.15 | 1.95 | 2.56 | 2.32 |
|  | 8 | 82 | 78 | 84 | 68 | 51 | 75 | 76 | 1.53 | 2.78 | 1.95 | -.22 | 3.48 | 1.37 | .65 | 2.32 | .21 |
|  | 9 | 84 | 74 | 58 | 72 | 60 | 85 | 61 | 1.23 | 1.95 | 2.32 | .88 | 3.48 | 2.32 | .43 | 5.15 | 1.95 |
|  | 10 | 80 | **56*** | 70 | 78 | **47*** | 86 | **54*** | 3.42 | 1.87 | 2.78 | 1.93 | 1.64 | 2.78 | 2.78 | 5.15 | 3.48 |
|  | 11 | 84 | 68 | **44*** | 74 | 57 | 90 | 74 | 1.09 | 1.61 | .88 | .50 | 2.71 | 1.25 | .29 | **.82*** | 2.78 |
|  | 12 | **64*** | **56*** | 56 | 66 | **33*** | 75 | 63 | **-.85*** | .43 | 1.37 | .88 | 1.11 | 1.64 | .18 | 1.37 | 1.11 |
|  | 13 | 76 | 64 | 76 | 66 | 56 | 75 | 74 | 1.47 | 3.48 | 3.48 | 3.48 | 3.48 | 3.48 | 2.32 | 3.48 | 2.78 |
|  | 14 | 98 | 86 | 78 | 82 | 76 | 86 | 92 | 2.34 | 3.48 | 2.78 | 2.78 | 2.78 | 2.32 | 3.48 | 1.64 | 1.95 |
|  | 15 | 90 | 92 | 86 | 88 | 82 | 83 | 90 | 3.11 | 2.32 | 2.32 | 1.95 | 3.48 | 3.48 | 1.37 | 3.48 | 2.78 |

CMT = Cambridge Memory Test, Ft = Face total, Ht = House total, Pt = Phone total; ON = Old/New tests; SD = standard deviation; ***** = **pathological** (z-score ≤ -2).

| **Stroke** | N° | **ArrayF** | **ArrayH** | **ArrayP** | **ArrayW** | **CMTFt** | **CMTHt** | **CMTPt** | **ONface** | **ONcar** | **ONflower** | **ONgun** | **ONhorse** | **ONhouse** | **ONglasses** | **ONtool** | **ONscene** |
| --- | --- | --- | --- | --- | --- | --- | --- | --- | --- | --- | --- | --- | --- | --- | --- | --- | --- |
| Right (n=13) | 16 | 82 | 74 | 66 | 76 | 60 | **64*** | 63 | 1.68 | 2.78 | 2.32 | 1.11 | 1.64 | 5.15 | 2.78 | 2.78 | 1.95 |
|  | 17 | 80 | 88 | 86 | 80 | 61 | 90 | 90 | 1.90 | 3.45 | 5.15 | 1.64 | 2.76 | 3.42 | 2.78 | 5.15 | 3.48 |
|  | 18 | 84 | 92 | 76 | 90 | 75 | 76 | 82 | 2.93 | 3.48 | 2.32 | 2.78 | 2.32 | 2.78 | 5.15 | 3.48 | 5.15 |
|  | 19 | **72*** | 66 | 62 | 72 | 61 | **64*** | 64 | 1.20 | 1.34 | 1.64 | .43 | **.15*** | 1.59 | .62 | 2.28 | 1.11 |
|  | 20 | 80 | 74 | 70 | 76 | 71 | 78 | 74 | **.75*** | 1.60 | 2.32 | 1.34 | 2.28 | 3.14 | 2.28 | 4.41 | 2.78 |
|  | 21 | **58*** | **48*** | 62 | **54*** | 61 | 69 | 64 | **-.04*** | 1.95 | 1.37 | 1.37 | 1.37 | 1.64 | 1.95 | 2.32 | 2.78 |
|  | 22 | 74 | **52*** | 58 | 64 | **47*** | **44*** | 65 | **-.25*** | **.17*** | .65 | -.08 | 2.15 | **-.22*** | -.22 | 2.78 | 5.15 |
|  | 23 | 80 | 68 | 64 | 88 | 71 | 74 | 81 | 2.93 | 1.37 | 1.95 | 2.32 | 2.32 | 5.15 | 2.78 | 1.64 | 1.37 |
|  | 24 | 78 | 76 | 58 | 70 | 63 | 74 | **57*** | 1.35 | 1.11 | 1.37 | 1.64 | **-.67*** | 1.34 | .65 | 1.93 | 1.65 |
|  | 25 | **62*** | **52*** | 66 | **56*** | **47*** | **49*** | **46*** | **-.30*** | .40 | 2.32 | -.22 | 1.95 | 1.25 | .43 | 2.15 | **-.44*** |
|  | 26 | **64*** | 66 | 58 | 62 | 53 | 72 | **58*** | .88 | .62 | 1.64 | .82 | .65 | 1.37 | .21 | 1.93 | 1.37 |
|  | 27 | **58*** | **56*** | 62 | 66 | **42*** | **51*** | 60 | - | - | - | - | - | - | - | - | - |
|  | 28 | 92 | 84 | 92 | 84 | 81 | 90 | 92 | 3.48 | 2.78 | 3.48 | 1.64 | 2.32 | 2.78 | 2.78 | 3.48 | 3.48 |

CMT = Cambridge Memory Test, Ft = Face total, Ht = House total, Pt = Phone total; ON = Old/New tests; SD = standard deviation; ***** = **pathological** (z-score ≤ -2).

| **Stroke** | N° | **ArrayF** | **ArrayH** | **ArrayP** | **ArrayW** | **CMTFt** | **CMTHt** | **CMTPt** | **ONface** | **ONcar** | **ONflower** | **ONgun** | **ONhorse** | **ONhouse** | **ONglasses** | **ONtool** | **ONscene** |
| --- | --- | --- | --- | --- | --- | --- | --- | --- | --- | --- | --- | --- | --- | --- | --- | --- | --- |
| Bilateral (n=3) | 29 | 86 | 74 | 70 | 78 | 61 | 78 | 78 | .93 | 1.91 | 1.37 | .43 | 3.45 | **.88*** | .65 | 5.15 | 1.95 |
|  | 30 | 86 | 64 | 60 | 68 | 60 | 78 | 82 | **.30*** | **.21*** | 1.95 | .65 | 1.95 | 2.78 | 1.95 | 1.64 | 1.11 |
|  | 31 | **70*** | 64 | 58 | 70 | 58 | **64*** | **53*** | **.13*** | **.21*** | .88 | 1.37 | .65 | 1.64 | .87 | 5.15 | 2.32 |
| **Controls** (n=41) | Mean | 86,34 | 77,27 | 78,29 | 81,56 | 75,27 | 83,13 | 77,98 | 2.39 | 3.02 | 2.63 | 1.89 | 2.81 | 3.75 | 2.2 | 3.28 | 3.09 |
|  | SD | 6,62 | 9,86 | 12,8 | 10,4 | 12,27 | 6,96 | 9,46 | .78 | 1.38 | 1.15 | 1.22 | 1.19 | 1.33 | 1.23 | 1.12 | 1.49 |
|  | Range | 74-100 | 56-94 | 56-100 | 60-100 | 50-94 | 67-94 | 51-94 | .32-4.08 | .43-5.15 | .65-5.15 | -.67-5.15 | 1.11-5.15 | 1.11-5.15 | .4-5.15 | 1.23-5.15 | .21-5.15 |

CMT = Cambridge Memory Test, Ft = Face total, Ht = House total, Pt = Phone total; ON = Old/New tests; SD = standard deviation; ***** = **pathological** (z-score ≤ -2).
