# Supplementary material for: Visual Agnosia and Posterior Cerebral Artery Infarcts: An Anatomical-Clinical Study
Source: PLoS One. 2012 Jan 20;7(1):e30433. doi: 10.1371/journal.pone.0030433 (PMC3262828; doi:10.1371/journal.pone.0030433)
Supplement: Table S3 — Reading test in 31 patients (and 41controls). (DOC) [file pone.0030433.s008.doc]

***Table 3*** *Reading test in 31 patients (and 41 controls)*

| **Stroke** | **N°** | **Reading (%)** | **Reading (TR msec)** | **Word length effect (msec/letter)** |
| --- | --- | --- | --- | --- |
| Left (n=15) | 1 | 99 | 861.45 | 31 |
|  | 2 | 99 | 970.12 | - .6 |
|  | 3 | 99 | 652.26 | 8.6 |
|  | 4 | 100 | 575.36 | 2.6 |
|  | 5 | 100 | 708.55 | 30.7 |
|  | 6 | 100 | 606.44 | 24.6 |
|  | 7 | 99 | 454.63 | 2 |
|  | 8 | **94*** | 834.32 | 62 |
|  | 9 | **95*** | 683.91 | 32 |
|  | 10 | 99 | 477.97 | 4.5 |
|  | 11 | 100 | 1039.44 | 32.9 |
|  | 12 | **86*** | 2014.3 | 314.8 |
|  | 13 | 99 | 955.18 | 105.8 |
|  | 14 | 100 | 1003.3 | 21.4 |
|  | 15 | 100 | 1056.1 | 27 |
| Right (n=13) | 16 | 99 | 618.7 | 7 |
|  | 17 | 100 | 459.94 | 6.1 |
|  | 18 | 100 | 465 | 1 |
|  | 19 | 99 | 705.47 | .39 |
|  | 20 | 99 | 568.26 | 3.4 |
|  | 21 | 100 | 721.34 | 40 |
|  | 22 | 100 | 719.41 | 4 |
|  | 23 | 99 | 555.93 | 4.4 |
|  | 24 | 100 | 714.16 | 15.4 |
|  | 25 | 98 | 890.12 | 52.8 |

SD = standard deviation; ***** = **pathological**.

| **Stroke** | **N°** | **Reading (%)** | **Reading (TR msec)** | **Word length effect (msec/letter)** |
| --- | --- | --- | --- | --- |
|  | 26 | 100 | 1230.9 | 23.54 |
|  | 27 | 100 | 775.05 | -4.9 |
|  | 28 | 100 | 1009.77 | 26.6 |
| Bilateral (n=3) | 29 | 100 | 514.34 | 14.7 |
|  | 30 | 100 | 760.95 | 29.8 |
|  | 31 | 99 | 816.59 | 12.5 |
| **Controls** (n=41) | Mean | 99,97 | 534.65 | -.48 |
|  | SD | 0,13 | 86.84 | 7.8 |
|  | Range | 99-100 | 431.8-755.32 | -22.5-24.2 |

SD = standard deviation; ***** = **pathological**.
