# Supplementary material for: Visual Agnosia and Posterior Cerebral Artery Infarcts: An Anatomical-Clinical Study
Source: PLoS One. 2012 Jan 20;7(1):e30433. doi: 10.1371/journal.pone.0030433 (PMC3262828; doi:10.1371/journal.pone.0030433)
Supplement: Table S4 — Results for CMT in 31 patients (and 41 controls). (DOC) [file pone.0030433.s009.doc]

***Table 4*** *Results for CMT in 31 patients (and 41 controls)*

|  |  | **CMTF** | | | | **CMTH** | | | | **CMTP** | | | |
| --- | --- | --- | --- | --- | --- | --- | --- | --- | --- | --- | --- | --- | --- |
| **Stroke** | **N°** | 1 | 2 | 3 | 2+3 | 1 | 2 | 3 | 2+3 | 1 | 2 | 3 | 2+3 |
| Left (n=15) | 1 | 100 | 77 | 75 | 76 | 89 | 50 | 54 | 52 | 89 | 83 | 79 | 81 |
|  | 2 | 89 | 50 | 38 | 44 | 89 | 67 | 83 | 74 | 100 | 80 | 71 | 76 |
|  | 3 | 100 | 97 | 88 | 93 | 100 | 77 | 88 | 81 | 94 | 83 | 79 | 81 |
|  | 4 | 100 | 93 | 71 | 83 | 94 | 90 | 88 | 89 | 89 | 90 | 67 | 80 |
|  | 5 | 100 | 60 | 54 | 57 | 100 | 87 | 71 | 80 | 100 | 70 | 75 | 72 |
|  | 6 | 94 | 77 | 71 | 74 | 89 | 93 | 83 | 89 | 89 | 70 | 71 | 70 |
|  | 7 | 83 | 77 | 71 | 74 | 100 | 93 | 92 | 93 | 100 | 77 | 75 | 76 |
|  | 8 | 78 | 47 | 38 | 43 | 94 | 63 | 75 | 69 | 89 | 77 | 67 | 72 |
|  | 9 | 89 | 60 | 38 | 50 | 100 | 77 | 83 | 80 | 78 | 53 | 58 | 56 |
|  | 10 | 100 | 37 | 21 | 30 | 100 | 87 | 75 | 81 | 72 | 47 | 50 | 48 |
|  | 11 | 89 | 43 | 50 | 46 | 100 | 93 | 79 | 87 | 94 | 67 | 67 | 67 |
|  | 12 | 67 | 17 | 29 | 22 | 100 | 73 | 58 | 67 | 83 | 60 | 50 | 56 |
|  | 13 | 89 | 53 | 33 | 44 | 94 | 63 | 75 | 69 | 83 | 73 | 67 | 70 |
|  | 14 | 100 | 90 | 42 | 69 | 94 | 90 | 75 | 83 | 100 | 87 | 92 | 89 |
|  | 15 | 100 | 83 | 67 | 76 | 89 | 80 | 83 | 81 | 100 | 93 | 79 | 87 |
| Right (n=13) | 16 | 94 | 60 | 33 | 48 | 89 | 67 | 42 | 56 | 94 | 67 | 33 | 52 |
|  | 17 | 94 | 50 | 50 | 50 | 94 | 87 | 92 | 89 | 100 | 87 | 88 | 87 |
|  | 18 | 100 | 80 | 50 | 67 | 100 | 73 | 63 | 69 | 94 | 77 | 79 | 78 |
|  | 19 | 78 | 60 | 50 | 56 | 94 | 53 | 54 | 54 | 89 | 57 | 54 | 56 |
|  | 20 | 100 | 67 | 54 | 61 | 94 | 70 | 75 | 72 | 100 | 70 | 58 | 65 |
|  | 21 | 89 | 60 | 42 | 52 | 100 | 50 | 71 | 59 | 78 | 57 | 63 | 59 |
|  | 22 | 56 | 43 | 46 | 44 | 39 | 50 | 42 | 46 | 78 | 60 | 63 | 61 |
|  | 23 | 100 | 77 | 42 | 61 | 94 | 63 | 71 | 67 | 94 | 83 | 67 | 76 |

CMT = Cambridge Memory Test, Ft = Face total, Ht = House total, Pt = Phone total; SD = standard deviation.

|  |  | **CMTF** | | | | **CMTH** | | | | **CMTP** | | | |
| --- | --- | --- | --- | --- | --- | --- | --- | --- | --- | --- | --- | --- | --- |
| **Stroke** | **N°** | 1 | 2 | 3 | 2+3 | 1 | 2 | 3 | 2+3 | 1 | 2 | 3 | 2+3 |
|  | 24 | 94 | 60 | 42 | 52 | 94 | 70 | 63 | 67 | 61 | 57 | 54 | 56 |
|  | 25 | 78 | 43 | 29 | 37 | 67 | 53 | 29 | 43 | 72 | 47 | 25 | 37 |
|  | 26 | 83 | 50 | 33 | 43 | 94 | 70 | 58 | 65 | 94 | 53 | 38 | 46 |
|  | 27 | 39 | 47 | 38 | 43 | 78 | 43 | 42 | 43 | 89 | 63 | 33 | 50 |
|  | 28 | 94 | 90 | 58 | 76 | 100 | 90 | 83 | 87 | 100 | 93 | 83 | 89 |
| Bilateral (n=3) | 29 | 94 | 50 | 50 | 50 | 100 | 73 | 67 | 70 | 94 | 87 | 54 | 72 |
|  | 30 | 94 | 63 | 29 | 48 | 89 | 77 | 71 | 74 | 100 | 80 | 71 | 76 |
|  | 31 | 100 | 50 | 38 | 44 | 83 | 57 | 58 | 57 | 78 | 40 | 50 | 44 |
| Controls | Mean | 96,48 | 73,09 | 62,09 | 68,20 | 97,02 | 80,98 | 75,41 | 78,50 | 93,63 | 75,93 | 68,80 | 72,76 |
|  | SD | 4,93 | 17,41 | 14,70 | 15,29 | 3,95 | 10,28 | 11,81 | 9,33 | 7,81 | 12,21 | 12,98 | 11,43 |
|  | Range | 83-100 | 33-97 | 38-88 | 37-93 | 89-100 | 53-97 | 46-96 | 56-93 | 72-100 | 43-100 | 38-92 | 44-93 |

CMT = Cambridge Memory Test, Ft = Face total, Ht = House total, Pt = Phone total; SD = standard deviation.
