# Supplementary material for: Visual Agnosia and Posterior Cerebral Artery Infarcts: An Anatomical-Clinical Study
Source: PLoS One. 2012 Jan 20;7(1):e30433. doi: 10.1371/journal.pone.0030433 (PMC3262828; doi:10.1371/journal.pone.0030433)
Supplement: Text S1 — Supplemental data. (DOC) [file pone.0030433.s010.doc]

### Visual agnosia and posterior cerebral artery infarcts: an anatomical-clinical study

Olivier Martinaud, MD (1, 2, 3,*), Dorothée Pouliquen, (1), Emmanuel Gérardin, MD, PhD (4), Maud Loubeyre, (1), David Hirsbein, MD (5), Didier Hannequin, MD (1, 6), Laurent Cohen, MD, PhD (2, 3, 7).

**Supplemental data**

We review briefly all patients with a significant, but not necessarily selective, deficit for a given category of stimuli, and consider whether deficits are associated with lesions affecting the corresponding specialized area (PPA, FFA, LOC, VWFA). With respect to each such area, lesions including the whole region of interest were considered as major lesions, while lesions encroaching or touching the region of interest were considered as minor lesions. In each category, we consider first patients with a deficit on the CMT (or on the timed reading test in the case of words), and then patients with an impairment on the Old/New or on the Array test.

**IMPAIRED PERCEPTION OF WORDS**

**Patients impaired on the timed reading test**

Three patients with left PCA strokes were impaired on the overt reading task (Supplemental Fig. 2).

Patient #8 had a small lesion (purple) involving the deep branches of the calcarine artery with a small lesion in the inferior part of the mesial occipital cortex. The lesion was thus clearly posterior to the lesion site associated to pure alexia [1,2]. We therefore checked the nature of this patient’s reading errors. All his 10 errors affected the rightmost part of stimuli (e.g. cigarette > cigare; sourire > sourit). Thus, considering that this patient had dense macular hemianopia, his errors likely resulted from hemianopic alexia rather than from pure alexia [1,3,4].

Patient #9’s stroke (green) associated the superficial branches of the calcarine artery and the temporal artery, with a large lesion of the posterior lateral and mesial part of the temporal cortex, encompassing the peak of the VWFA. It is possible that the absence of a significant word length effect was due to the long delay elapsed between the stroke and the present evaluation (> 18 months). Indeed, rehabilitation has been shown to progressively erase the word length effect in pure alexic patients [5,6,7]. Some evidence of neural reorganization bilaterally in the posterior fusiform could contribute to word reading after brain damage [1,8].

Patient #12’s lesion (yellow) involved most of the territory of the PCA, sparing its most lateral part, and also including the peak of the VWFA. This patient had the most typical features of mild pure alexia, including a significant word length effect. Note that he had associated deficits on the Array tests for faces and houses, the CMT with faces and the Old/New test for faces. Accordingly, his lesion was extensive enough to affect also the PPA and FFA.

**Patients impaired on other tests**

A fourth patient (#6) had no difficulties with the reading task, but showed a selective deficit on the Array test for words. His lesion (blue) involved the territory of the deep branches of the calcarine artery, affecting the mesial ventral temporal cortex, contiguous to the peak of the VWFA.

Two further patients (#21 and #25) were impaired on several Array tests (words, faces and houses). Their lesions were right-hemispheric, and the deficit probably resulted from a non-specific visuo-spatial deficit affecting all detection tasks. Thus, patient #21 had a pathological score on the PEGV, and patient #25 on the Navon test, a pattern suggestive of simultanagnosia.

**Patients with no deficit**

Two patients (#1 and #5) had a lesion in the left temporal cortex just in contact with the VWFA, and no impairment with words. The fact that the lesion spared most of the region of interest may explain this normal performance.

All the other patients showed no difficulties with words, and their lesions spared the VWFA entirely.

In summary, for 3 out of 4 patients, impaired word processing could be attributed to the overlap of the lesion with the VWFA: two major lesions (#9 and #12) were responsible of alexia, one minor lesion (#6) resulted in a deficit on the Array test only, and two minor lesions (#1 and #5) caused no impairment.

**IMPAIRED PERCEPTION OF HOUSES**

**Patients impaired on the CMT with houses**

Seven patients were impaired on the CMT with houses (Supplemental Fig. 3).

Patient #1 had a haemorrhagic left PCA stroke (blue), with a lesion just posterior to the average peak of the PPA. Patient #16 had a right PCA stroke (orange) with a very small occipital lesion, posterior to the PPA. Patient #19 had a right PCA stroke (purple) with a mesial occipito-temporal lesion, extending anteriorily to the peak of the PPA. Patients #22 (green), #25 (yellow) and #27 (cyan) had an extensive right PCA stroke with a mesial occipito-temporal lesion encompassing the peak of the PPA. Patient #31 had a bilateral mesial occipito-temporal stroke (brown), including the right PPA and sparing the left PPA. These 5 last patients had several other deficits, which probably resulted from the wide extension of their lesions: the lesion of patient #19 also affected the OFA, the lesion of patients #22 and #27 also included the OFA and the FFA, and the lesion of patients #25 and #31 included also the OFA, the FFA and the LOC.

**Patients impaired on other tests**

Patient #29 had no difficulties on the CMT with houses, but showed a selective deficit on the Old/New test for houses. He had a bilateral PCA stroke with right predominant mesial temporal lesions including the peak of the PPA on both sides. Although this patient had no memory deficit on the standardized tests, this result could be due to a memory deficit rather than to actual agnosia. One possible account is that memory retrieval mechanisms for houses depends mostly on the activity of the retrosplenial cortex (RSC) [9,10]. Accordingly, the lesion of patient #29 was located very close to the two RSC, which might suggest that the deficit result from a disconnection of the RSC. However, there is no explanation for the absence of agnosia, while the lesion of patient #29 included the two PPA.

Finally, patients #12 and #21 were impaired non-selectively on the Array test for houses. They had, respectively, a left-hemispheric lesion including the PPA, and a right-hemispheric lesion overlapping with the PPA. Patients #12 and 21 had difficulties also with faces and several other types of tests, which could be due to the extension of their lesions.

## Patients with no deficit

Conversely, the lesion of 9 out of the 20 patients who had no difficulties for houses included or overlapped with the PPA region of interest, in the left hemisphere for 4 patients (#3, 5, 6, 9) and in the right hemisphere for 5 patients (#17, 20, 23, 24, 26).

In summary, anatomical-clinical correlations are less clear-cut for houses than for words. An impairment could be associated with left, right, or bilateral lesions, with no significant asymmetry, even if right lesions tended to be associated with a slightly higher risk of deficit: among the 6 lesions affecting only the left PPA, 2 (33%) induced a deficit, while among the 11 lesions affecting only the right PPA, 6 induced a deficit (55%). One minor left lesion (#1), one minor right lesion (#19), and 4 major right lesions (#22, #25, #27, #31) were responsible of a deficit on the CMT with houses. One major left lesion (#12), and one minor right lesion (#21) caused a deficit on the Array test for houses.

**IMPAIRED PERCEPTION OF FACES**

**Patients impaired on the CMT with faces**

Five patients were impaired on the CMT with faces (Supplemental Fig. 4).

Patient #10 had a left PCA stroke (blue) involving the deep branches of the calcarine artery with a lesion of the mesial part of the ventral occipital cortex, posterior to the peak of the FFA and sparing the OFA. The lesions of patients #12 (yellow), 22 (green), 25 (purple) and 27 (cyan), already described, included or overlapped the FFA or the OFA, on the left side for the first patient and the right for the three others.

## Patients impaired on other tests

The lesion of patient #19, who showed a selective deficit on the Array test for faces, has been described before. It was just posterior and superior to the peak of the right FFA, and included the OFA. Patient #20 who showed a selective deficit on the Old/New test for faces had a right mesial occipito-temporal lesion sparing both the FFA and the OFA. Patient #21 who showed a selective deficit on the Old/New test for faces had a right large occipito-temporal lesion, including both the FFA and the OFA. Patient #26 who showed a selective deficit on the Array test for faces had a right PCA stroke with a lesion of the mesial temporal cortex extending forward and including the FFA, but not the OFA. The lesion of patient #31 who showed a selective deficit on the Array test and on the Old/New test for faces has been already described. It spared the left FFA and the left OFA, but included the right FFA and OFA.

Note that no pure deficit for faces was identified: out of the 10 patients with a pathological score on the Array test, the CMT or the Old/New test with faces, 9 had some other visual deficit, especially with houses. Accordingly, the lesions of 8 patients were extensive enough to affect also the PPA. Patient #10 is the only one to have several deficits, on the Array test for houses and the CMT with faces and phones, without lesion of the FFA, the PPA or the LOC. She had no difficulties in several tasks (DO80, the Doors test and the recognition test from the Benton visual retention test), which are usually impaired in case of integrative agnosia. Moreover on the Navon task, and contrary to patient HJA, our patient was impaired at processing visual stimuli at a global rather than a local level [11]. Indeed, this unexpected pattern likely resulted from simultanagnosia.

**Patients with no deficit**

The lesion of 6 patients with no difficulties for faces included or overlapped with the peak of the FFA, on the left side for 5 patients (#1, 3, 5, 6, 9) and on both sides for one patient (#29).

In summary, right lesions tend to be associated with a higher risk of deficit for faces: among the 6 lesions affecting only the left FFA, only one (17%) induced a deficit, while all the 6 lesions affecting only the right FFA induced a deficit (100%). One major left lesion (#12), 2 minor right lesions (#22, #27), and 1 major right lesion (#25) were responsible of a deficit on the CMT with faces. One major right lesion (#26) caused a deficit on the Array test for faces, one major right lesion (#21) resulted in a deficit on the Old/New test for faces and one major right lesion (#31) resulted in a deficit on the two tests.

**IMPAIRED PERCEPTION OF PHONES**

**Patients impaired on the CMT with phones**

Five patients were impaired on the CMT with phones (Supplemental Fig. 5).

The lesion (blue) of patient #10 (see above) was located mesial to the left LOC. Patient #24 had a similar right-hemispheric lesion (green), mesial to the right LOC. The lesion (yellow) of patient #25 (see above) was slightly overlapping with the right LOC. Patient #26 had a right PCA stroke (purple) with a lesion of the mesial ventral temporal cortex, anterior and mesial to the LOC. The lesion (cyan) of patient #31 (see above) was contiguous to the right LOC.

## Patients impaired on other tests

Patients #2 and 11 had no difficulties on the CMT with phones, but showed a selective deficit on the Array test for phones. Patient #2 had a left PCA stroke with a very small lesion of the mesial occipital cortex posterior to the LOC. Patient #11 had a similar but somewhat larger lesion as compared to patient #2.

The LOC is mostly supplied by the middle cerebral artery, and hence generally spared in PCA infarcts. Hence only 2 right- and 2 left-hemispheric lesions affected the LOC. Although only the 2 right-sided lesions were associated with a deficit, no safe conclusions can be drawn from so small a dataset.

# REFERENCES

1. Cohen L, Martinaud O, Lemer C, Lehericy S, Samson Y, et al. (2003) Visual word recognition in the left and right hemispheres: anatomical and functional correlates of peripheral alexias. Cereb Cortex 13: 1313-1333.

2. Pflugshaupt T, Gutbrod K, Wurtz P, von Wartburg R, Nyffeler T, et al. (2009) About the role of visual field defects in pure alexia. Brain 132: 1907-1917.

3. Leff AP, Crewes H, Plant GT, Scott SK, Kennard C, et al. (2001) The functional anatomy of single-word reading in patients with hemianopic and pure alexia. Brain 124: 510-521.

4. Leff AP, Spitsyna G, Plant GT, Wise RJ (2006) Structural anatomy of pure and hemianopic alexia. J Neurol Neurosurg Psychiatry 77: 1004-1007.

5. Behrmann M, Black SE, Bub D (1990) The evolution of pure alexia: a longitudinal study of recovery. Brain Lang 39: 405-427.

6. Cohen L, Henry C, Dehaene S, Martinaud O, Lehericy S, et al. (2004) The pathophysiology of letter-by-letter reading. Neuropsychologia 42: 1768-1780.

7. Gaillard R, Naccache L, Pinel P, Clemenceau S, Volle E, et al. (2006) Direct intracranial, FMRI, and lesion evidence for the causal role of left inferotemporal cortex in reading. Neuron 50: 191-204.

8. Tsapkini K, Vindiola M, Rapp B Patterns of brain reorganization subsequent to left fusiform damage: fMRI evidence from visual processing of words and pseudowords, faces and objects. Neuroimage.

9. Epstein RA, Parker WE, Feiler AM (2007) Where am I now? Distinct roles for parahippocampal and retrosplenial cortices in place recognition. J Neurosci 27: 6141-6149.

10. Epstein RA (2008) Parahippocampal and retrosplenial contributions to human spatial navigation. Trends Cogn Sci 12: 388-396.

11. Riddoch MJ, Humphreys GW, Akhtar N, Allen H, Bracewell RM, et al. (2008) A tale of two agnosias: distinctions between form and integrative agnosia. Cogn Neuropsychol 25: 56-92.
